# Association of the planetary health diet score with obesity, high blood pressure, dyslipidemia, and cardiometabolic risk markers: Using data from the 2016–2020 Korea National Health and Nutrition Examination Survey

**DOI:** 10.1371/journal.pone.0350821

**Published:** 2026-06-08

**Authors:** Ujin Lee, Eugene Kang, Bomi Kim, Diana Shubekova, Adiyasuren Dookhuu, Heejin Lee, Jung Eun Lee, Minji Kang

**Affiliations:** 1 Department of Food and Nutrition, Duksung Women’s University, Seoul, South Korea; 2 Department of Pharmacy, Duksung Women’s University, Seoul, South Korea; 3 Department of Food and Nutrition, Seoul National University, Seoul, South Korea; 4 Department of Nutrition, Harvard T.H. Chan School of Public Health, Boston, Massachusetts, United States of America; Barking Havering and Redbridge Hospitals NHS Trust: Barking Havering and Redbridge University Hospitals NHS Trust, UNITED KINGDOM OF GREAT BRITAIN AND NORTHERN IRELAND

## Abstract

Noncommunicable diseases (NCDs) remain the leading cause of death worldwide, with unhealthy diets and insufficient physical activity as major contributing factors. The Planetary Health Diet is a sustainable dietary pattern linked to chronic diseases, but studies in Asian populations remain limited. Therefore, the aim of this study was to investigate the associations between the Planetary Health Diet score and cardiometabolic risk factors among Korean adults. We used KNHANES 2016–2020 data (n = 25,336), and the Planetary Health Diet score was calculated in accordance with the Lancet Commission guidelines. Participants were categorized into quartiles according to the Planetary Health Diet score, and multivariable logistic regression models were used to examine associations with obesity, hypertension, dyslipidemia, an elevated triglyceride-to-HDL cholesterol ratio, impaired fasting glucose, and an elevated HbA1c level. A higher Planetary Health Diet score was significantly associated with lower odds of hypercholesterolemia in women and was related to reduced prevalence of abdominal obesity, hypertension, low HDL-cholesterolemia, and elevated glycated hemoglobin (HbA1c) depending on age group. No significant associations were observed in men. These findings suggest that higher Planetary Health Diet scores may be associated with selected cardiometabolic markers among Korean women. Further research is warranted to confirm the applicability of the Planetary Health Diet score in Korean dietary contexts.

## Introduction

Noncommunicable diseases (NCDs), also known as chronic diseases, are long-term conditions caused by a combination of genetic, physiological, environmental, and behavioral factors [1]. According to the World Health Organization (WHO), NCDs cause 41 million deaths each year, accounting for 74% of all deaths globally, and in the Republic of Korea, 77% of all deaths in 2021 were attributable to NCDs [2,3]. With the rapidly aging population, the burden of chronic diseases is expected to continue to increase. Major metabolic risk factors—including hypertension, obesity, dyslipidemia, and diabetes—substantially increase the likelihood of developing NCDs such as cardiovascular disease [1]. These metabolic abnormalities, including elevated blood pressure, high blood glucose, excess body weight, and abnormal lipid profiles, are becoming increasingly common worldwide. Therefore, the effective management of metabolic risk factors is essential for reducing the burden of NCDs. Some modern dietary habits, particularly diets high in fat, sugar, and salt, adversely affect health and contribute to the development of chronic diseases. Traditionally, the Korean diet is low in fat and calories, characterized by high rice and vegetable intake [4]. However, the modification of traditional diets toward Westernized dietary patterns has led to an increase in the consumption of high-fat and high-calorie foods, becoming a major cause of chronic diseases [5,6]. The increasing intake of sugar-sweetened beverages and processed foods is associated with higher rates of obesity and diabetes [7]. Moreover, the steady increase in meat consumption has heightened the risk of colorectal cancer and cardiovascular diseases [8,9]. Conversely, the inadequate consumption of fruits, vegetables, and whole grains is associated with a lack of dietary fiber and micronutrients, potentially contributing to vitamin and mineral deficiencies as well as digestive problems [10]. These dietary changes in Korea have become significant contributors to NCDs. Therefore, reducing the consumption of salt and sugar while increasing the intake of fruits, vegetables, whole grains, and fiber-rich foods is crucial for preventing NCDs. A sustainable diet simultaneously protects human health and the global environment by minimizing negative environmental impacts while efficiently utilizing resources to ensure access to safe and nutritious food for both current and future generations [11]. The Planetary Health Diet offers a multidimensional dietary approach that considers both health and environmental factors, and the Planetary Health Diet score serves as an important tool to encourage shifts toward healthier and more sustainable dietary patterns, contributing to the achievement of the UN Sustainable Development Goals (SDGs) and the Paris Climate Agreement targets [12]. In 2019, the Lancet Commission on Planetary Health proposed the Planetary Health Diet as a sustainable dietary pattern designed to promote human health while remaining within environmental limits. Compared with standard Western dietary patterns, the diet emphasizes higher fruit, vegetable, plant-based protein, and unsaturated fat intake, and reduced red meats consumption [12]. The Planetary Health Diet, designed to promote human health and environmental sustainability, includes daily recommendations such as 500 g of fruits and vegetables, 232 g of whole grains, 50 g of nuts, 75 g of legumes, and moderate amounts of animal protein (14 g of red meat, 29 g of poultry, and 28 g of fish); additionally, salt and sugar intake are reduced, and the consumption of healthy fats (40 g of unsaturated fats) is recommended. The aim of this diet is to prevent NCDs and protect the environment, necessitating changes in food production, policies, and consumer behaviors. Previous studies have investigated the associations between sustainable dietary indices and chronic diseases. A Danish and Swedish cohort study revealed that higher adherence to sustainable dietary indices was associated with a lower incidence of diabetes [13–15]. In the NutriNet-Santé cohort in France, compared with the highest-adherence group, the group with the lowest adherence to a sustainable dietary pattern had a higher initial body weight and greater weight gain rates [16].The DONALD cohort study in Germany also revealed significant negative correlations between adolescent sustainable diet compliance and adult anthropometric indicators, including weight, BMI, and waist circumference [17]. However, some studies report no significant associations between sustainable dietary indices and cardiovascular disease [18]. The aim of this study was to investigate the associations between the Planetary Health Diet score and metabolic risk factors, including obesity, hypertension, dyslipidemia, an elevated triglyceride-to-HDL cholesterol ratio, impaired fasting glucose, and an elevated HbA1c level, among Korean adults.

## Methods

### Study design and participants

In this study, data from the 7th (2016–2018) and the 1st and 2nd years (2019–2020) of the 8th Korean National Health and Nutrition Examination Survey (KNHANES, 2016–2020) were utilized. The study included healthy adults aged 19 years and older who participated in all three components of the survey: health interview, medical examination, and nutrition survey. A total of 25,336 participants were selected after excluding individuals with a history of underlying medical conditions (n = 4,476), such as stroke, myocardial infarction and angina, diabetes, gastric cancer, liver cancer, colon cancer, breast cancer, cervical cancer, lung cancer, and thyroid cancer; individuals without physical or biochemical data (n = 2,513); and pregnant women (n = 78). Participants with missing values for the main exposure and outcome variables were excluded from the analysis. Missing values for selected covariates were handled using simple single-category substitution based on prespecified categories; this approach was applied to education level (n = 1,273), household income (n = 80), smoking status (n = 207), monthly drinking frequency (n = 193), and physical activity practice (n = 1,301). The final sample consisted of 10,630 males and 14,706 females. The use of KNHANES 2016–2020 data was approved by the Korean Institutional Review Board (IRB approval numbers: 2018-01-03-P-A for 2018, 2018-01-03-C-A for 2019, and 2018-01-03-2C-A for 2020).

### Dietary assessment and planetary health diet score calculation

The dietary data used in this study were obtained from the food intake survey of the Korean National Health and Nutrition Examination Survey (KNHANES); the data were collected via a 24-hour dietary recall method conducted by trained interviewers during face-to-face household visits. The 24-hour dietary recall included detailed information on meals (meal classification, time, location, meal availability, and presence of companions) and ingested foods (food name, amount consumed, and food codes) consumed in the 24 hours prior to the survey. To increase accuracy, volume calculations and assistive tools such as food containers, food models, measuring cups and spoons, thickeners, 30 cm rulers, and tape measures were employed. Sustainable dietary patterns in this study were evaluated using the Planetary Health Diet score, which was developed by Knuppel et al. based on the EAT-Lancet recommendations [19]. The Planetary Health Diet score comprises 14 dietary components across 9 categories (Table A in S1 Table): whole grains (e.g., rice, grains, and corn; ≤ 464 g/day and whole grain fiber >5 g) tubers and starchy vegetables (potatoes; and cassava; ≤ 100 g/day); vegetables (all vegetables; ≥ 200 g/day); fruits (all fruits; ≥ 100 g/day), dairy products (whole milk or equivalents; ≤ 500 g/day), protein sources (beef, lamb, and pork: ≤ 28 g/day; chicken; and other poultry: ≤ 58 g/day; eggs: ≤ 25 g/day; and fish: ≤ 100 g/day); legumes (dry beans, lentils, and peas: ≤ 100 g/day; soy foods: ≤ 50 g/day; and peanuts and tree nuts: ≥ 25 g/day); added fats (palm oil, unsaturated oils, and dairy fats including milk, lard or tallow, with a ratio of 0.8 for unsaturated to saturated fat intake); and added sugars (all sweeteners; ≤ 31 g/day). The cutoff values for dietary components were adopted from the scoring criteria proposed by Knuppel et al. based on the EAT-Lancet reference diet [19]. Adherence to each component was scored as 1 point, and nonadherence was scored as 0 points, yielding a total score ranging from 0 to 14. This approach was based on the scoring method used in a previous study [20]. A higher Planetary Health Diet score indicated better adherence to a healthy and sustainable diet. The total diet scores were categorized into quartiles for analysis.

### Anthropometric and biochemical assessments

Body mass index (BMI) was calculated as weight in kilograms divided by the square of height in meters (kg/m²) to assess obesity status. BMI was categorized as follows: normal weight (BMI < 23 kg/m²), overweight (23 ≤ BMI < 25 kg/m²), and obesity (BMI ≥ 25 kg/m²). Waist circumference was measured to assess abdominal obese using the Korean Obesity Society’s cutoffs of ≥90 cm for men and ≥85 cm for women. Hypertension was defined as systolic blood pressure ≥130 mmHg or diastolic blood pressure ≥85 mmHg. The following dyslipidemia-related indicators were assessed using the indicated cutoffs: elevated total cholesterol (>200 mg/dL), elevated triglycerides (TG; > 150 mg/dL), low HDL cholesterol (<40 mg/dL for men and <50 mg/dL for women), and elevated LDL cholesterol (>130 mg/dL). In addition, the TG/HDL ratio (considered increased if ≥3), fasting glucose (considered impaired if fasting blood glucose (FBG) ≥100 mg/dL), and glycated hemoglobin (HbA1c) level (considered elevated if ≥5.7%) were analyzed.

### Covariates assessments and odds ratios

Demographic and lifestyle factors were analyzed using the following variables: age, sex, marital status, household income, education level, smoking status, alcohol consumption, physical activity, and current menstrual status. The following variables were considered covariates because of their potential to confound the associations between a sustainable diet and metabolic risk factors: marital status (married, unmarried), household income (low, medium–low, medium–high, high), education level (middle/high school or less, high school, university or above), smoking status (nonsmoker, former smoker, current smoker), alcohol consumption at least once a month (no, yes), physical activity (no, yes), and body mass index categories (normal weight, overweight, obese).

### Statistical analysis

We accounted for the complex sample design of the KNHANES data, incorporating weights, stratification variables, and cluster variables. All analyses were conducted separately by sex, and age was categorized into three groups: 19–39, 40–64, and 65 years or older. The Rao‒Scott chi-square test was used to assess differences across quartiles of the Planetary Health Diet score, with analyses performed separately for men and women. Associations between the Planetary Health Diet score and health-related factors were examined using linear regression, and least-squares means (LS-means) and 95% confidence intervals (CIs) were estimated. Two models were used for adjustment: Model 1 adjusted for age (year, continuous) and energy intake (kilocalories, continuous) and Model 2 was additionally adjusted for household income (low, middle-low, middle-high, high), smoking status (never, past, ever), marital status (married, unmarried), education level (elementary school or below, middle school, high school, college or above), prevalence of monthly alcohol use (No, Yes), and physical activity (no, yes). For women, menopause status was also included. Logistic regression models were used to assess the associations between the Planetary Health Diet score and the incidence of obesity, hypertension, dyslipidemia, an elevated TG/HDL ratio, impaired fasting glucose, and an elevated HbA1c level. Odds ratios (ORs) and 95% confidence intervals (CIs) were estimated using the same covariates included in the linear regression models. To evaluate linear trends, the median value of each quartile of the Planetary Health Diet score was modeled as a continuous variable. The quartile cutoffs for the score were 0–7 (reference), 8, 9, 10, and 11–14 points. All the statistical analyses were performed using SAS version 9.4 (SAS Institute, Cary, NC, USA), and statistical significance was defined as p < 0.05.

## Results

### General characteristics

Table 1 presents the characteristics of the male participants (n = 10,630) according to Planetary Health Diet score quartiles. The mean (SE) age and total energy intake were 44.7 (0.2) years and 2,342.2 (12.3) kcal, respectively. Among men, 51.4% had a college degree or higher, 71.5% consumed alcohol at least once per month, 33.8% were current smokers, and 48.6% were physically active. With respect to body weight status, 25.4% of the men were overweight, and 42.6% were obese. Men with higher Planetary Health Diet scores tended to be older, have lower energy intake, be more likely to be married and physically active, and be less likely to be overweight or obese. Table 2 summarizes the characteristics of the female participants (n = 14,706) by Planetary Health Diet score quartile. The mean (SE) age and total energy intake were 46.8 (0.2) years and 1,647.4 (7.4) kcal, respectively. Among women, 45.6% held a college degree or higher, 78.8% were married, 32.9% had a high household income, 46.8% reported monthly alcohol consumption, 5.8% were current smokers, and 42.5% were physically active. Overall, 18.8% were overweight, and 26.3% were obese. Women with higher Planetary Health Diet scores tended to be older, have lower energy intake, consume less alcohol, be married, be nonsmokers, and be menopausal.

**Table 1:** Characteristics of study participants according to the Planetary Health Diet Score Quartiles in men.

		Planetary Health Diet Score quartiles				p value
	All (n = 10,630)	Q1 (n = 2,949)	Q2 (n = 2,823)	Q3 (n = 2,703)	Q4 (n = 2,155)	
	mean ± s.e.					
Age (years)	44.7 ± 0.2	39.1 ± 0.3	43.5 ± 0.3	47.1 ± 0.4	53.3 ± 0.5	<.0001
Energy intake (kcal)	2,342.1 ± 12.3	2,649.6 ± 22.1	2,333.8 ± 22.5	2,164.8 ± 18.9	2,048.1 ± 19.8	<.0001
	% (s.e.)					
Age group						<.0001
19-39	41.2 (0.7)	55.7 (1.1)	43.7 (1.1)	33.9 (1.1)	21.4 (1.2)	
40-64	46.1 (0.6)	40.2 (1.0)	45.7 (1.1)	50.7 (1.1)	50.9 (1.3)	
65 or more	12.7 (0.4)	4.1 (0.3)	10.6 (0.6)	15.4 (0.7)	27.7 (1.1)	
Education level						<.0001
Middle school or below	12.4 (0.4)	7.0 (0.5)	10.3 (0.6)	14.9 (0.8)	21.9 (1.0)	
High school	36.2 (0.6)	39.3 (1.1)	35.6 (1.1)	35.3 (1.1)	33.0 (1.2)	
College or above	51.4 (0.7)	53.7 (1.1)	54.1 (1.2)	49.9 (1.2)	45.0 (1.4)	
Marital status						<.0001
Married	67.8 (0.7)	58.7 (1.2)	66.5 (1.1)	71.5 (1.1)	81.3 (1.1)	
Unmarried	32.2 (0.7)	41.3 (1.2)	33.5 (1.1)	28.5 (1.1)	18.7 (1.1)	
Household income						<.0001
Low	11.7 (0.4)	8.4 (0.6)	9.9 (0.7)	13.1 (0.8)	18.5 (1.0)	
Middle-low	21.8 (0.5)	20.9 (0.8)	22.2 (1.0)	21.5 (1.0)	23.0 (1.1)	
Middle-high	30.0 (0.6)	31.7 (1.0)	30.0 (1.1)	29.3 (1.1)	28.0 (1.2)	
High	36.5 (0.8)	38.9 (1.1)	37.9 (1.2)	36.1 (1.3)	30.5 (1.3)	
Smoking status						<.0001
Never smokers	32.6 (0.5)	34.0 (1.0)	33.1 (1.0)	31.0 (1.0)	31.1 (1.2)	
Former-smokers	33.6 (0.5)	27.1 (0.9)	32.3 (1.0)	37.2 (1.1)	42.3 (1.3)	
Current smokers	33.8 (0.6)	38.8 (1.0)	34.6 (1.1)	31.7 (1.0)	26.6 (1.2)	
Prevalence of monthly alcohol use (≥1/month)						<.0001
No	28.5 (0.5)	26.5 (0.9)	26.8 (1.0)	28.5 (1.0)	34.9 (1.3)	
Yes	71.5 (0.5)	73.5 (0.9)	73.2 (1.0)	71.5 (1.0)	65.1 (1.3)	
^a^Physical activity						0.0001
No	51.4 (0.6)	49.1 (1.1)	49.6 (1.1)	53.9 (1.2)	55.1 (1.3)	
Yes	48.6 (0.6)	50.9 (1.1)	50.4 (1.1)	46.1 (1.2)	44.9 (1.3)	
Body mass index (kg/m^2^)						0.0118
Normal weight (BMI < 23)	32.0 (0.5)	31.2 (1.0)	31.2 (1.0)	31.7 (1.0)	33.4 (1.2)	
Over weight (23 ≤ BMI < 25)	25.4 (0.5)	23.7 (0.8)	25.5 (1.0)	25.6 (1.0)	28.3 (1.1)	
Obesity (25 ≤ BMI)	42.6 (0.6)	44.1 (1.0)	43.2 (1.1)	42.7 (1.2)	38.3 (1.2)	

^a^Physical activity: Individuals who engaged in at least 2 hours and 30 minutes of moderate-intensity physical activity, or 1 hour and 15 minutes of vigorous-intensity physical activity, or an equivalent combination of both (with 1 minute of vigorous activity corresponding to 2 minutes of moderate activity) per week.

**Table 2:** Characteristics of study participants according to the Planetary Health Diet Score Quartiles in women.

		Planetary Health Diet Score quartiles				p value
	All (n = 14,706)	Q1 (n = 2,964)	Q2 (n = 3,723)	Q3 (n = 4,158)	Q4 (n = 3,861)	
	mean ± s.e.					
Age (years)	46.8 ± 0.2	39.8 ± 0.3	44.4 ± 0.3	48.7 ± 0.3	54.0 ± 0.3	<.0001
Energy intake (kcal)	1,647.4 ± 7.4	1,831.1 ± 17.0	1,633.0 ± 13.3	1,570.7 ± 12.7	1,575.3 ± 11.4	
	% (s.e.)					
Age group						<.0001
19-39	36.0 (0.5)	54.1 (1.0)	41.5 (1.0)	30.9 (0.9)	18.3 (0.8)	
40-64	48.1 (0.5)	38.8 (1.0)	45.9 (1.0)	51.5 (0.9)	55.8 (1.0)	
65 or more	15.9 (0.4)	7.1 (0.5)	12.6 (0.6)	17.6 (0.7)	25.9 (0.8)	
Education level						<.0001
Middle school or below	20.9 (0.5)	10.3 (0.6)	17.2 (0.7)	23.1 (0.8)	32.6 (0.9)	
High school	33.5 (0.5)	35.7 (1.1)	35.1 (0.9)	33.0 (0.9)	30.2 (0.9)	
College or above	45.6 (0.7)	54.0 (1.2)	47.7 (1.1)	43.8 (1.0)	37.1 (1.1)	
Marital status						<.0001
Married	78.8 (0.5)	66.0 (1.1)	76.1 (0.9)	82.8 (0.8)	89.6 (0.7)	
Unmarried	21.2 (0.5)	34.0 (1.1)	23.9 (0.9)	17.2 (0.8)	10.4 (0.7)	
Household income						<.0001
Low	14.3 (0.5)	9.1 (0.6)	13.1 (0.7)	15.3 (0.7)	19.5 (0.8)	
Middle-low	23.7 (0.5)	22.9 (1.0)	23.3 (0.9)	23.8 (0.8)	25.0 (0.9)	
Middle-high	29.1 (0.5)	30.5 (1.0)	30.8 (0.9)	28.7 (0.9)	26.2 (0.9)	
High	32.9 (0.7)	37.5 (1.2)	32.9 (1.1)	32.2 (1.0)	29.4 (1.0)	
Smoking status						<.0001
Never smokers	89.1 (0.3)	86.4 (0.8)	88.5 (0.6)	89.5 (0.6)	91.9 (0.6)	
Former-smokers	5.1 (0.2)	6.3 (0.5)	5.0 (0.4)	5.2 (0.4)	3.8 (0.4)	
Current smokers	5.8 (0.3)	7.2 (0.6)	6.6 (0.5)	5.2 (0.5)	4.3 (0.4)	
Prevalence of monthly alcohol use (≥1/month)						<.0001
No	53.2 (0.5)	45.2 (1.0)	50.8 (1.0)	54.5 (0.9)	62.2 (1.0)	
Yes	46.8 (0.5)	54.8 (1.0)	49.2 (1.0)	45.5 (0.9)	37.8 (1.0)	
^a^Physical activity						<.0001
No	57.5 (0.5)	53.1 (1.0)	57.8 (1.0)	60.0 (1.0)	58.4 (1.0)	
Yes	42.5 (0.5)	46.9 (1.0)	42.2 (1.0)	40.0 (1.0)	41.6 (1.0)	
Menopause status						<.0001
No	64.0 (0.5)	79.3 (0.8)	70.5 (0.9)	60.1 (0.9)	46.6 (1.0)	
Yes	36.0 (0.5)	20.7 (0.8)	29.5 (0.9)	39.9 (0.9)	53.4 (1.0)	
Body mass index (kg/m^2^)						<.0001
Normal weight (BMI < 23)	54.8 (0.5)	59.7 (1.0)	57.9 (0.9)	52.9 (0.9)	49.0 (1.0)	
Over weight (23 ≤ BMI < 25)	18.8 (0.4)	16.3 (0.7)	17.6 (0.7)	19.9 (0.7)	21.6 (0.8)	
Obesity (25 ≤ BMI)	26.3 (0.5)	24.1 (0.9)	24.6 (0.8)	27.3 (0.8)	29.4 (0.9)	

^a^Physical activity: Individuals who engaged in at least 2 hours and 30 minutes of moderate-intensity physical activity, or 1 hour and 15 minutes of vigorous-intensity physical activity, or an equivalent combination of both (with 1 minute of vigorous activity corresponding to 2 minutes of moderate activity) per week.

### Meeting Planetary Health Diet Component Criteria According to Score Quartiles

The distribution of adherence to individual Planetary Health Diet recommendations among men is shown in Table 3. Participants with lower Planetary Health Diet scores were more likely to consume less than the recommended amounts of rice, wheat, and other grains (≤464 g/day, whole grain fiber >5 g), vegetables (≥200 g/day), fruits (≥100 g/day), red meat (beef, lamb, pork: ≤ 28 g/day), poultry (≤58 g/day), eggs (≤25 g/day), fish (≤100 g/day), soy foods (≤50 g/day), and added sugars (≤31 g/day). Table 4 presents the adherence patterns among women. Female participants were more likely to fall within the higher quartiles of the Planetary Health Diet score. As the adherence scores increased, the proportion of participants who met the Planetary Health Diet recommendations for each food group also increased. Compared with men, women demonstrated greater adherence to fruit, dairy product, protein source, soy food, and added sugar recommendations. However, men consumed more whole grains, starchy vegetables, and added fats. No significant sex differences were observed in the intake of dry beans, lentils, peanuts or nuts.

**Table 3:** Proportion of participants meeting each Planetary Health Diet Score criterion in men.

		Planetary Health Diet Score quartiles					p value
Dietary components	Criteria for scoring 1 point	All (n = 10,630)	Q1 (n = 2,949)	Q2 (n = 2,823)	Q3 (n = 2,703)	Q4 (n = 2,155)	
		mean ± s.e.					
Planetary Health Diet Score	range 0–14 points	9.2 (0.02)*	7.5 (0.01)	9.0 (0.0)	10.0 (0.0)	11.3 (0.01)	<.0001
		% (s.e.)					
Whole grains							
1. Rice, wheat, corn, and other	≤464g/day and whole grain fiber > 5g	36.8 (0.6)*	18.2 (0.8)	30.6 (1.0)	46.2 (1.1)	67.3 (1.3)	<.0001
Tubers and starchy vegetables							
2. Potatoes and cassava	≤100g/day	90.4 (0.4)*	81.5 (0.8)	92.3 (0.6)	95.0 (0.4)	96.7 (0.4)	<.0001
Vegetables							
3. All vegetables	≥200g/day	73.1 (0.5)*	61.4 (1.0)	69.8 (1.1)	80.4 (0.9)	89.4 (0.8)	<.0001
Fruits							
4. All fruits	≥100g/day	36.2 (0.6)*	17.4 (0.8)	30.0 (1.0)	44.2 (1.1)	68.8 (1.2)	<.0001
Dairy foods							
5. Whole milk or derivative equivalents	≤500g/day	98.4 (0.2)*	97.1 (0.4)	98.0 (0.3)	99.6 (0.1)	99.8 (0.1)	<.0001
Protein sources							
6. Beef, lamb, pork	≤28g/day	31.0 (0.5)*	15.9 (0.7)	27.1 (0.9)	36.4 (1.1)	57.3 (1.3)	<.0001
7. Chicken, other poultry	≤58g/day	81.9 (0.4)*	67.7 (1.0)	83.5 (0.9)	89.9 (0.7)	93.7 (0.6)	<.0001
8. Eggs	≤25g/day	59.8 (0.6)*	40.6 (1.0)	58.9 (1.1)	69.6 (1.0)	82.1 (0.9)	<.0001
9. Fish	≤100g/day	59.1 (0.6)*	42.4 (1.1)	58.6 (1.1)	67.6 (1.0)	78.1 (1.0)	<.0001
Legumes							
10. Dry beans, lentils, peas	≤100g/day	99.9 (0.03)	99.9 (0.0)	99.8 (0.1)	100 (0.0)	100 (0.0)	0.0586
11. Soy foods	≤50g/day	84.1 (0.4)*	74.0 (0.9)	85.0 (0.8)	89.0 (0.7)	94.1 (0.6)	<.0001
12. Peanuts or tree nuts	≥25g/day	4.4 (0.2)	1.2 (0.2)	2.9 (0.3)	4.9 (0.5)	11.9 (0.8)	<.0001
Added fats							
13. Palm oil, unsaturated oils, dairy fats (incl. in milk), lard or tallow	Ratio of 0.8 for unsaturated:saturated fat intake	97.7 (0.2)*	96.9 (0.3)	97.6 (0.3)	97.9 (0.3)	99.4 (0.2)	<.0001
Added sugars							
14. All sweeteners	≤31g/day	64.7 (0.6)*	37.4 (1.0)	65.8 (1.1)	79.3 (0.9)	91.6 (0.7)	<.0001

**Table 4:** Proportion of participants meeting each Planetary Health Diet Score criterion in women.

		Planetary Health Diet Score quartiles					*p* value
Dietary components	Criteria for scoring 1 point	All (n = 14,706)	Q1 (n = 2,964)	Q2 (n = 3,723)	Q3 (n = 4,158)	Q4 (n = 3,861)	
		mean ± s.e.					
Planetary Health Diet Score	range 0–14 points	9.5 (0.01)*	7.6 (0.01)	9.0 (0.0)	10.0 (0.0)	11.3 (0.01)	<.0001
		% (s.e.)					
Whole grains							
1. Rice, wheat, corn, and other	≤464g/day and whole grain fiber > 5g	27.6 (0.5)*	9.6 (0.6)	19.2 (0.8)	28.5 (0.9)	53.6 (1.0)	<.0001
Tubers and starchy vegetables							
2. Potatoes and cassava	≤100g/day	89.0 (0.3)*	80.1 (0.9)	87.4 (0.6)	92.5 (0.5)	95.4 (0.4)	<.0001
Vegetables							
3. All vegetables	≥200g/day	58.4 (0.5)*	37.9 (1.1)	52.0 (1.0)	61.9 (0.9)	81.2 (0.7)	<.0001
Fruits							
4. All fruits	≥100g/day	47.0 (0.6)*	21.9 (0.9)	36.3 (1.0)	53.5 (1.0)	75.9 (0.9)	<.0001
Dairy foods							
5. Whole milk or derivative equivalents	≤500g/day	98.8 (0.1)*	97.2 (0.3)	99.0 (0.2)	99.3 (0.1)	99.6 (0.2)	<.0001
Protein sources							
6. Beef, lamb, pork	≤28g/day	45.6 (0.5)*	24.6 (0.9)	38.1 (0.9)	50.3 (0.9)	69.0 (0.9)	<.0001
7. Chicken, other poulty	≤58g/day	87.3 (0.3)*	74.0 (1.0)	86.7 (0.6)	91.5 (0.5)	95.8 (0.4)	<.0001
8. Eggs	≤25g/day	63.0 (0.5)*	44.0 (1.1)	57.8 (0.9)	68.3 (0.9)	81.1 (0.8)	<.0001
							(Continued)
9. Fish	≤100g/day	69.7 (0.5)*	53.6 (1.1)	64.3 (0.9)	75.5 (0.7)	84.7 (0.7)	<.0001
Legumes							
10. Dry beans, lentils, peas	≤100g/day	99.9 (0.03)	99.8 (0.1)	100 (0.0)	99.8 (0.1)	100 (0.0)	0.1607
11. Soy foods	≤50g/day	87.5 (0.3)*	78.0 (0.9)	86.1 (0.6)	91.2 (0.5)	94.0 (0.4)	<.0001
12. Peanuts or tree nuts	≥25g/day	4.3 (0.2)	0.9 (0.2)	2.3 (0.3)	3.6 (0.4)	10.6 (0.6)	<.0001
Added fats							
13. Palm oil, unsaturated oils, dairy fats (incl. in milk), lard or tallow	Ratio of 0.8 for unsaturated:saturated fat intake	96.8 (0.2)*	93.4 (0.5)	96.3 (0.3)	97.8 (0.3)	99.4 (0.1)	<.0001
Added sugars							
14. All sweeteners	≤31g/day	75.7 (0.5)*	45.4 (1.1)	74.6 (0.9)	86.3 (0.6)	94.0 (0.5)	<.0001

### Least-Squares Means of Health Indicators According to Planetary Health Diet Score

Table 5 presents the least-squares means (LS-means) of health indicators according to quartiles of the Planetary Health Diet score among men. BMI, waist circumference, systolic and diastolic blood pressure, total cholesterol, high-density lipoprotein (HDL) cholesterol, the TG/HDL ratio, FBG, and HbA1c level were not significantly associated with the Planetary Health Diet score quartile. Across age groups, no consistent associations were observed between the Planetary Health Diet score and cardiometabolic indicators (Tables A–C in S2 Table). In Model 2, for men aged 40–64 years, a higher Planetary Health Diet score was significantly associated with a lower waist circumference (p for trend = 0.0281). In Model 1, among those aged ≥ 65 years, BMI increased modestly with higher Planetary Health Diet scores (p for trend = 0.0301), although the association was not significant in Model 2. Table 6 presents the LS means of the health indicators according to Planetary Health Diet score quartiles for women. The Planetary Health Diet score was significantly associated with HDL cholesterol level, the TG/HDL ratio, and HbA1c level. In Model 1, the HDL cholesterol concentration decreased and the TG/HDL ratio increased significantly with increasing score (p for trend = 0.0089 and 0.0171, respectively). After further adjustments in Model 2, higher Planetary Health Diet scores were associated with lower HDL cholesterol and HbA1c levels but a higher TG/HDL ratio. Age-stratified analyses (Table D-F in S2 Table) revealed that among women aged 19–39 years, higher Planetary Health Diet scores were associated with lower BMI and waist circumference (p for trend < 0.05). In Models 1 and 2, BMI and waist circumference decreased significantly as the Planetary Health Diet score increased. In the 40–64 age group, higher scores were associated with lower HDL and total cholesterol levels and a higher TG/HDL ratio. In Model 1, as the Planetary Health Diet score increased, total cholesterol and high-density lipoprotein (HDL) cholesterol levels decreased, and the TG/HDL ratio increased; the results were the same in Model 2. Among those aged ≥65 years, higher Planetary Health Diet scores were related to lower systolic and diastolic blood pressure and FBG level but higher a TG/HDL ratio. In Model 1, higher Planetary Health Diet scores were associated with low diastolic blood pressure, low high-density lipoprotein (HDL) cholesterol and high fasting blood glucose (FBG) levels and a high triglyceride (TG) level and triglyceride (TG)/high-density lipoprotein (HDL) ratio; in Model 2, the associations were similar to those in Model 1, but FBG was not significant even though there was a trend toward a decrease.

**Table 5:** Least-squares means (95% confidence intervals) of health outcome according to the Quartiles of Planetary Health Diet Score in men.

	Quartiles of the Planetary Health Diet Score				
	Q1	Q2	Q3	Q4	*p* for trend
LS-means (95% CIs)	(n = 2,949)	(n = 2,823)	(n = 2,703)	(n = 2,155)	
**Body mass index (kg/m²)**					
model 1	24.64 (24.48-24.79)	24.72 (24.57-24.87)	24.70 (24.55-24.86)	24.52 (24.35-24.70)	0.5570
model 2	24.59 (24.43-24.75)	24.65 (24.49-24.81)	24.65 (24.48-24.82)	24.50 (24.32-24.69)	0.6811
**Waist circumferences (cm)**					
model 1	86.91 (86.50-87.31)	86.89 (86.48-87.30)	86.76 (86.33-87.19)	86.35 (85.84-86.86)	0.1260
model 2	86.71 (86.28-87.14)	86.65 (86.21-87.09)	86.56 (86.10-87.02)	86.28 (85.74-86.82)	0.2259
**Systolic blood pressure (mmHg)**					
model 1	119.57 (119.02-120.12)	120.27 (119.69-120.86)	120.00 (119.38-120.62)	119.49 (118.78-120.19)	0.9877
model 2	119.40 (118.82-119.98)	120.08 (119.45-120.71)	119.72 (119.10-120.34)	119.19 (118.45-119.92)	0.7218
**Diastolic blood pressure (mmHg)**					
model 1	78.26 (77.83-78.70)	78.70 (78.25-79.16)	78.49 (78.00-78.98)	77.97 (77.46-78.48)	0.5245
model 2	77.86 (77.39-78.34)	78.25 (77.76-78.74)	78.12 (77.61-78.62)	77.86 (77.34-78.38)	0.9084
**Triglyceride (mg/dL)**					
model 1	157.21 (151.49-162.92)	154.60 (149.34-159.86)	159.91 (153.68-166.14)	155.99 (148.04-163.94)	0.8541
model 2	147.32 (141.71-152.93)	145.30 (139.79-150.81)	151.72 (145.49-157.94)	151.51 (143.23-159.79)	0.2243
**Hight-density lipoprotein (mg/dL)**					
model 1	47.82 (47.35-48.29)	48.51 (48.03-49.00)	48.48 (47.98-48.97)	47.89 (47.31-48.48)	0.5520
model 2	47.61 (47.12-48.09)	48.17 (47.68-48.67)	48.09 (47.57-48.61)	47.52 (46.91-48.12)	0.9152
**Total cholesterol (mg/dL)**					
model 1	194.45 (192.96-195.94)	195.67 (194.14-197.20)	194.50 (192.96-196.04)	192.81 (190.80-194.81)	0.2253
model 2	192.62 (191.05-194.19)	193.73 (192.14-195.32)	193.02 (191.46-194.59)	192.20 (190.24-194.16)	0.7662
**TG/HDL**					
model 1	3.73 (3.55-3.90)	3.61 (3.44-3.79)	3.75 (3.56-3.95)	3.80 (3.53-4.08)	0.5372
model 2	3.51 (3.33-3.68)	3.42 (3.23-3.61)	3.59 (3.40-3.79)	3.74 (3.44-4.03)	0.1297
**Fasting blood glucose (mg/dL)**					
model 1	98.67 (97.97-99.37)	99.02 (98.27-99.77)	99.31 (98.51-100.11)	99.09 (98.08-100.10)	0.3616
model 2	98.30 (97.57-99.02)	98.68 (97.88-99.48)	99.01 (98.12-99.89)	98.97 (97.94-100.00)	0.2018
**HbA1c (%)**					
model 1	5.58 (5.56-5.60)	5.59 (5.56-5.61)	5.58 (5.55-5.60)	5.57 (5.53-5.60)	0.4922
model 2	5.57 (5.55-5.60)	5.58 (5.55-5.61)	5.57 (5.54-5.60)	5.57 (5.53-5.60)	0.7440

Abbreviations: LS-means, least-squares means; CIs, confidence intervals; Q, quartile; TG/HDL, triglyceride to HDL-cholesterol ratio: HbA1c, hemoglobin A_1C_. model 1 adjusted for age (year, continuous) and energy intake (kcal, continuous) model 2 additionally adjusted for household income (low, middle-low, middle-high, high), smoking status (never, past, ever), marriage status (married, unmarried), education level (elementary school or below, middle school, high school, college or above), prevalence of monthly alcohol use(No, Yes), physical activity (No, Yes).

**Table 6:** Least-squares means (95% confidence intervals) of health outcome according to the Quartiles of Planetary Health Diet Score in women.

	Quartiles of the Planetary Health Diet Score				
	Q1	Q2	Q3	Q4	p for trend
LS-means (95% CIs)	(n = 2,964)	(n = 3,723)	(n = 4,158)	(n = 3,861)	
**Body mass index (kg/m²)**					
model 1	23.24 (23.06-23.41)	23.11 (22.98-23.25)	23.11 (22.97-23.25)	23.12 (22.97-23.28)	0.3709
model 2	23.26 (23.09-23.43)	23.10 (22.98-23.23)	23.11 (22.97-23.25)	23.11 (22.97-23.26)	0.2418
**Waist circumferences (cm)**					
model 1	78.39 (77.98-78.79)	78.13 (77.79-78.48)	78.19 (77.83-78.55)	78.12 (77.73-78.51)	0.4186
model 2	78.46 (78.06-78.85)	78.09 (77.76-78.42)	78.18 (77.83-78.52)	78.11 (77.73-78.49)	0.2975
**Systolic blood pressure (mmHg)**					
model 1	114.80 (114.21-115.40)	114.24 (113.72-114.77)	114.58 (114.07-115.09)	114.26 (113.67-114.84)	0.3360
model 2	114.74 (114.17-115.32)	114.28 (113.77-114.78)	114.66 (114.15-115.16)	114.19 (113.62-114.77)	0.3354
**Diastolic blood pressure (mmHg)**					
model 1	73.43 (73.02-73.83)	73.27 (72.89-73.64)	73.49 (73.16-73.82)	73.33 (72.97-73.69)	0.9638
model 2	73.47 (73.07-73.87)	73.26 (72.88-73.63)	73.46 (73.13-73.79)	73.33 (72.97-73.69)	0.8221
**Triglyceride (mg/dL)**					
model 1	105.58 (103.13-108.03)	107.39 (104.49-110.30)	106.21 (103.69-108.73)	109.73 (106.83-112.63)	0.0821
model 2	105.64 (103.22-108.07)	107.21 (104.30-110.11)	106.23 (103.74-108.73)	109.85 (106.96-112.73)	0.0691
**Hight-density lipoprotein (mg/dL)**					
model 1	56.48 (55.95-57.01)	56.50 (56.02-56.97)	56.37 (55.91-56.82)	55.50 (55.00-56.00)	0.0089
model 2	56.36 (55.84-56.88)	56.54 (56.08-57.00)	56.35 (55.92-56.79)	55.58 (55.09-56.07)	0.0263
**Total cholesterol (mg/dL)**					
model 1	195.18 (193.75-196.60)	195.55 (194.19-196.90)	195.57 (194.31-196.83)	194.86 (193.38-196.35)	0.7868
model 2	195.29 (193.87-196.71)	195.63 (194.30-196.97)	195.43 (194.20-196.67)	194.82 (193.33-196.30)	0.6302
**TG/HDL**					
model 1	2.10 (2.03-2.16)	2.15 (2.06-2.23)	2.13 (2.06-2.19)	2.25 (2.16-2.34)	0.0171
model 2	2.10 (2.04-2.17)	2.14 (2.06-2.23)	2.13 (2.06-2.19)	2.25 (2.16-2.33)	0.0174
**Fasting blood glucose (mg/dL)**					
model 1	95.04 (94.45-95.63)	94.98 (94.49-95.46)	95.06 (94.59-95.53)	94.71 (94.19-95.24)	0.4813
model 2	95.07 (94.49-95.65)	94.94 (94.45-95.42)	95.04 (94.58-95.51)	94.75 (94.23-95.27)	0.5037
**HbA1c (%)**					
model 1	5.56 (5.53-5.58)	5.54 (5.52-5.55)	5.54 (5.52-5.55)	5.53 (5.51-5.55)	0.0806
model 2	5.56 (5.54-5.58)	5.54 (5.52-5.55)	5.54 (5.52-5.55)	5.53 (5.51-5.54)	0.0393

Abbreviations: LS-means, least-squares means; CIs, confidence intervals; Q, quartile; TG/HDL, triglyceride to HDL-cholesterol ratio: HbA1c, hemoglobin A1C. model 1 adjusted for age (year, continuous) and energy intake (kcal, continuous) model 2 additionally adjusted for household income (low, middle-low, middle-high, high), smoking status (never, past, ever), marriage status (married, unmarried), education level (elementary school or below, middle school, high school, college or above), prevalence of monthly alcohol use(No, Yes), physical activity (No, Yes), menopause status (No, Yes).

### Odds Ratios of Health Indicators According to the Planetary Health Diet Score

Tables 7 and 8 present the associations between Planetary Health Diet score quartiles and the prevalence of obesity, dyslipidemia, hypertension, an elevated TG/HDL ratio, impaired fasting glucose, and elevated HbA1c levels. Among men (Table 7), no significant associations were observed between Planetary Health Diet score quartiles and the odds of obesity, dyslipidemia, hypertension, an elevated TG/HDL ratio, impaired fasting glucose, or elevated HbA1c level across all the models (p for trend > 0.05). Among women (Table 8), higher Planetary Health Diet scores were inversely associated with the incidence of dyslipidemia. In Model 2, compared with women in the lowest quartile, women in the highest quartile had 13% lower odds having of dyslipidemia (OR = 0.87, 95% CI: 0.77–1.00; p for trend = 0.0393). In Model 2, the odds ratio for the risk of a high HbA1c level was 0.87, but the decreasing trend was not significant; additionally, there was no association with other indicators. According to the results of the age-stratified analyses (Table A-F in Table S3), women aged 19–39 years with higher Planetary Health Diet scores had significantly lower odds of having abdominal obesity (OR = 0.75, 95% CI: 0.58–0.98; p for trend = 0.0329) and hypertension (OR = 0.43, 95% CI: 0.25–0.74; p for trend = 0.0117). Among those aged 40–64 years, higher Planetary Health Diet scores were associated with lower odds of hypercholesterolemia (OR = 0.79, 95% CI: 0.67–0.93; p for trend = 0.0115) and a high HbA1c level (OR = 0.83, 95% CI: 0.70–1.00; p for trend = 0.0494). In women aged ≥65 years, higher Planetary Health Diet scores were related to lower odds of hypertension (OR = 0.61, 95% CI: 0.46–0.80; p for trend = 0.0119) and impaired FBG (OR = 0.73, 95% CI: 0.56–0.96; p for trend = 0.0227) but higher odds of an elevated TG/HDL ratio (OR = 1.29, 95% CI: 0.93–1.78; p for trend = 0.0305). No other significant associations were observed between the adherence to the Planetary Health Diet and health outcomes among women. For men, no significant associations were observed between Planetary Health Diet adherence and health outcomes across any of the assessed outcomes.

**Table 7:** Odds ratios (95% confidence intervals) of health outcome according to the Quartiles of Planetary Health Diet Score in men.

	ORs (95% CIs) according to the quartiles Planetary Health Diet Score				
	Q1	Q2	Q3	Q4	*p* for trend
ORs (95% CIs)	(n = 2,949)	(n = 2,823)	(n = 2,703)	(n = 2,155)	
**Body mass index (≥25 kg/m²)**					
model 1	1	1.00 (0.89-1.13)	1.01 (0.89-1.16)	0.89 (0.77-1.01)	0.1975
model 2	1	0.99 (0.88-1.12)	1.02 (0.89-1.16)	0.90 (0.79-1.04)	0.3175
**Waist circumferences (≥90 cm)**					
model 1	1	0.97 (0.86-1.09)	0.99 (0.87-1.12)	0.92 (0.80-1.06)	0.3507
model 2	1	0.97 (0.86-1.09)	0.99 (0.87-1.13)	0.94 (0.82-1.09)	0.5444
**Blood pressure (Systolic blood pressure ≥130 mmHg or Diastolic blood pressure ≥85 mmHg)**					
model 1	1	1.07 (0.94-1.21)	1.03 (0.90-1.17)	0.89 (0.77-1.03)	0.1905
model 2	1	1.06 (0.93-1.21)	1.02 (0.90-1.16)	0.91 (0.78-1.05)	0.2401
**Triglyceride (≥150 mg/dL)**					
model 1	1	0.98 (0.86-1.11)	1.03 (0.91-1.18)	0.91 (0.79-1.05)	0.4565
model 2	1	0.98 (0.86-1.12)	1.06 (0.93-1.21)	0.99 (0.86-1.15)	0.7058
**High-density lipoprotein (<40mg/dL)**					
model 1	1	0.95 (0.83-1.10)	0.91 (0.79-1.05)	0.99 (0.84-1.16)	0.6001
model 2	1	0.98 (0.85-1.13)	0.94 (0.81-1.08)	1.04 (0.88-1.22)	0.9502
**Low-density lipoprotein (≥130 mg/dL)**					
model 1	1	0.99 (0.87-1.13)	0.98 (0.86-1.13)	0.93 (0.80-1.08)	0.3787
model 2	1	0.99 (0.87-1.13)	1.00 (0.87-1.14)	0.95 (0.82-1.11)	0.6090
**Total cholesterol (≥200 mg/dL)**					
model 1	1	1.04 (0.92-1.17)	1.05 (0.92-1.20)	0.93 (0.80-1.07)	0.5422
model 2	1	1.03 (0.92-1.17)	1.07 (0.93-1.22)	0.98 (0.84-1.14)	0.9354
**TG/HDL (≥3)**					
model 1	1	1.02 (0.90-1.16)	1.02 (0.90-1.16)	0.94 (0.82-1.08)	0.5212
model 2	1	1.03 (0.91-1.17)	1.06 (0.93-1.19)	1.02 (0.88-1.17)	0.6369
**Fasting blood glucose (≥100 mg/dL)**					
model 1	1	1.06 (0.93-1.20)	1.06 (0.93-1.21)	1.04 (0.89-1.20)	0.5582
model 2	1	1.06 (0.93-1.21)	1.07 (0.93-1.22)	1.09 (0.93-1.26)	0.2754
**HbA1c (≥5.7%)**					
model 1	1	0.96 (0.84-1.10)	0.97 (0.84-1.11)	0.88 (0.76-1.03)	0.1495
model 2	1	0.97 (0.85-1.12)	0.99 (0.86-1.13)	0.92 (0.79-1.07)	0.3535

Abbreviations: ORs, odds ratios; CIs, confidence intervals; Q, quartile; TG/HDL, triglyceride to HDL-cholesterol ratio: HbA1c, hemoglobin A1C. Model 1 adjusted for age (year, continuous) and energy intake (kcal, continuous) model 2 additionally adjusted for household income (low, middle-low, middle-high, high), smoking status (never, past, ever), marriage status (married, unmarried), education level (elementary school or below, middle school, high school, college or above), prevalence of monthly alcohol use(No, Yes), physical activity (No, Yes).

**Table 8:** Odds ratios (95% confidence intervals) of health outcome according to the Quartiles of Planetary Health Diet Score in women.

	ORs (95% CLs) according to the quartiles Planetary Health Diet Score				
	Q1	Q2	Q3	Q4	p for trend
ORs (95% CIs)	(n = 2,964)	(n = 3,723)	(n = 4,158)	(n = 3,861)	
**Body mass index (≥25 kg/m²)**					
model 1	1	0.92 (0.80-1.04)	0.96 (0.84-1.09)	0.96 (0.84-1.09)	0.7292
model 2	1	0.90 (0.79-1.03)	0.95 (0.83-1.08)	0.94 (0.82-1.07)	0.5610
**Waist circumferences (≥90 cm)**					
model 1	1	0.94 (0.82-1.07)	0.94 (0.82-1.08)	0.94 (0.81-1.08)	0.4486
model 2	1	0.91 (0.80-1.05)	0.92 (0.80-1.06)	0.92 (0.79-1.06)	0.3247
**Blood pressure (Systolic blood pressure ≥130 mmHg or Diastolic blood pressure ≥85 mmHg)**					
model 1	1	0.94 (0.81-1.10)	0.98 (0.84-1.13)	0.88 (0.76-1.01)	0.1151
model 2	1	0.94 (0.81-1.09)	0.98 (0.84-1.14)	0.87 (0.76-1.01)	0.0964
**Triglyceride (≥150 mg/dL)**					
model 1	1	1.03 (0.88-1.21)	0.98 (0.83-1.14)	1.11 (0.95-1.30)	0.2835
model 2	1	1.01 (0.86-1.19)	0.96 (0.82-1.12)	1.10 (0.94-1.28)	0.3332
**High-density lipoprotein (<50mg/dL)**					
model 1	1	0.99 (0.88-1.12)	1.01 (0.90-1.14)	1.11 (0.99-1.26)	0.0735
model 2	1	0.98 (0.87-1.10)	1.00 (0.89-1.13)	1.09 (0.97-1.24)	0.1329
**Low-density lipoprotein (≥130 mg/dL)**					
model 1	1	1.01 (0.89-1.14)	1.04 (0.93-1.18)	0.97 (0.85-1.10)	0.7498
model 2	1	1.00 (0.88-1.13)	1.02 (0.91-1.16)	0.94 (0.83-1.07)	0.4661
**Total cholesterol (≥200 mg/dL)**					
model 1	1	0.97 (0.87-1.10)	0.96 (0.86-1.08)	0.89 (0.78-1.01)	0.0658
model 2	1	0.97 (0.86-1.09)	0.95 (0.84-1.06)	0.87 (0.77-1.00)	0.0393
**TG/HDL (≥3)**					
model 1	1	0.96 (0.82-1.11)	1.00 (0.86-1.15)	1.12 (0.97-1.30)	0.0626
model 2	1	0.93 (0.80-1.09)	0.98 (0.84-1.14)	1.10 (0.95-1.28)	0.0950
**Fasting blood glucose (≥100 mg/dL)**					
model 1	1	1.04 (0.90-1.21)	1.03 (0.90-1.19)	1.01 (0.88-1.17)	0.9341
model 2	1	1.02 (0.88-1.18)	1.01 (0.88-1.16)	1.00 (0.87-1.16)	0.9825
**HbA1c (≥5.7%)**					
model 1	1	0.92 (0.80-1.05)	0.94 (0.83-1.07)	0.89 (0.77-1.02)	0.1530
model 2	1	0.91 (0.79-1.04)	0.93 (0.81-1.06)	0.87 (0.75-0.99)	0.0739

Abbreviations: CIs, confidence intervals; HbA1c, hemoglobin A1c; ORs, odds ratios; Q, quartile; TG/HDL, triglyceride to HDL-cholesterol ratio. model 1 adjusted for age (year, continuous) and energy intake (kcal, continuous) model 2 additionally adjusted for household income (low, middle-low, middle-high, high), smoking status (never, past, ever), marriage status (married, unmarried), education level (elementary school or below, middle school, high school, college or above), prevalence of monthly alcohol use(No, Yes), physical activity (No, Yes), menopause status (No, Yes).

## Discussion

In this study, we examined the associations between the Planetary Health Diet score and various health outcomes. Sex-stratification analyses were conducted for obesity, hypertension, dyslipidemia, an elevated TG/HDL ratio, impaired fasting glucose, and an elevated HbA1c level. For men, the average Planetary Health Diet score was 9.2, and for women, it was 9.5. Higher Planetary Health Diet scores were associated with a greater proportion of men who were aged 40 years or older, were married, had less energy intake, were nonsmokers, and had a BMI ≥ 25 kg/m². In addition, individuals with higher adherence tended to be older and more likely to have a diet score above the median; adults aged ≥40 years were less likely to be current smokers than younger adults were. Among men, the Planetary Health Diet score was not significantly associated with obesity, hypertension, dyslipidemia, an elevated TG/HDL ratio, impaired fasting glucose, or an elevated HbA1c level. Among women, higher Planetary Health Diet scores were associated with older age, being married, not smoking, not drinking, and menopausal status. In women, higher adherence to the Planetary Health Diet was also associated with a lower incidence of hypercholesterolemia, whereas low HDL-cholesterolemia tended to be more prevalent among those with higher adherence.

Sex-stratified analyses of adherence to the Planetary Health Diet and related sociodemographic and lifestyle factors among Korean adults revealed patterns consistent with those observed in previous studies [13–15,21–24]. In a companion analysis of Korean adults using the same Planetary Health Diet scoring framework, women had higher total scores than men did, and adherence was found to be differentially associated with sociodemographic and lifestyle factors according to sex [20]. Specifically, older age was associated with higher adherence among both sexes; furthermore, widowhood and being a current smoker were associated with lower adherence among men, and a never-married status and monthly alcohol use were associated with lower adherence among women. Similar findings were observed in the Mexican Teachers’ Cohort, in which adherence to a sustainable dietary pattern varied according to menopausal status [22]. Evidence from studies assessing the Korea Healthy Eating Index (KHEI) among Korean adults also supports the trends observed in the present study [25]. High KHEI adherence was associated with older age, being a women, and having high income and education levels. Moreover, the prevalence of obesity tended to decrease with increasing adherence, whereas being a current smoking and consuming alcohol more than once per month were inversely correlated [25]. Taken together, these sex-specific adherence patterns may provide additional context for interpreting the more apparent associations with cardiometabolic markers observed among women in the present study.

Among both men and women, older age was consistently associated with Planetary Health Diet scores that were above the median. Among men, current smoking status was associated with scores below the median, whereas among women, lower-than-median scores were observed among those who had a high school-level of education or lower, were unmarried, had a high income, and were nonmenopausal. When the data were stratified by age, current smoking status was associated with lower-than-median scores for both men and women aged 40 years and older. Among men aged 19–39 years, normal weight was associated with lower-than-median scores, and among men aged 40–64 years, university-level education or above was associated with lower adhence to the Planetary Health Diet. Among women aged 19–39 years, normal weight and an unmarried status were associated with lower-than-median scores, and among women aged 40–64 years, a high school education or higher and menstrual status were associated with lower-than-median scores. However, among women aged 40–64 years, abstaining from alcohol consumption (drinking less than once per month) was associated with scores above the median.

Analysis of the associations between the Planetary Health Diet score and sociodemographic and lifestyle characteristics revealed patterns similar to those reported in previous studies [26–29]. A prior multiethnic cohort study examining dietary quality indicators revealed that higher diet quality was associated with older age, higher education, physical activity, and multivitamin use, corresponding to scores above the median, whereas overweight or obesity, being a current smoking, and heavy alcohol consumption were associated with lower-than-median scores among both men and women [29]. Additionally, being widowed, underweight, and a former smoker were associated with diet scores lower than the median among men but not among women. International studies conducted in Australia, Brazil, and Europe have also demonstrated that diet quality is positively associated with older age, higher education, and non-married status, whereas smoking and obesity are negatively associated [26–28].

Among male participants, no significant associations were observed between adherence to the Planetary Health Diet and health outcomes. When the data were stratified by age, no significant associations were found for men aged 19–39 years. Among men aged 40–64 years, the prevalence of high blood pressure was lower in the second quartile than in the first quartile of the diet score; however, no significant trend was identified across increasing adherence levels. Among men aged 65 years or older, the prevalence of obesity was lower in the second quartile than in the first quartile, although the decrease was not significant across higher adherence categories. With respect to hypercholesterolemia, the prevalence was higher in the second quartile than in the first quartile, but no significant linear trend was observed. The sex-specific patterns observed in this study may reflect a combination of biological differences and potential differences in dietary reporting accuracy between men and women. One review suggested that sex differences in metabolic responses to diet may be influenced by hormonal differences, body composition, and reproductive physiological changes such as the menstrual cycle and menopause [30]. In addition, a previous study in the Korean population reported that the pattern of metabolic risk factors differs by sex, suggesting that such differences may have partly contributed to the sex-specific findings observed in this study [31]. Furthermore, a validation study among older Korean adults suggested that women may report food items more accurately than men do in 24-hour dietary recalls [32]. These biological, metabolic, and methodological factors may have acted in combination, potentially contributing to the absence of significant associations among men in the present study.

The different patterns observed among men compared with those reported in previous studies are likely attributable to four methodological factors. First, many earlier studies analyzed diet quality without stratification by sex, which may obscure sex-specific associations [13–15,19,22,23]. Second, differences in the calculation of sustainable diet indices across countries limit the comparability of findings. Although many indices are based on the EAT-Lancet reference diet, each index has been adapted to national dietary patterns and researcher discretion, resulting in methodological variation [33]. In this study, we employed the Planetary Health Diet score developed by Knuppel et al., whereas other studies used indices such as the Planetary Health Diet Index (PHDI) [23,34] and the Sustainable Diet Index (SDI) [16,35]. These indices differ in both their included food groups and scoring criteria. For instance, the Brazilian PHDI assigns 5–10 points to 16 dietary components, yielding a total score of 0–150 points [33]. The SDI, developed in France based on FAO definitions, includes four subindices—nutritional, environmental, economic, and sociocultural—and produces a total score of 4–20 points by assigning 1–5 points per dimension [33]. Third, the method of dietary data collection varies across studies. In this study, the dietary index was calculated using 24-hour recall data, whereas other indices rely on food frequency questionnaires (FFQs). Fourth, previous studies have primarily examined the associations between sustainable diet indices and chronic diseases rather than intermediate health indicators. Because this study focused on metabolic risk factors rather than diagnosed diseases, direct comparisons with earlier findings are limited.

Among all female participants, higher Planetary Health Diet scores were associated with a lower incidence of hypercholesterolemia (p for trend = 0.0393). Among women aged 19–39 years, higher Planetary Health Diet scores were associated with a lower incidence of abdominal obesity and hypertension (p for trend = 0.0329; p for trend = 0.0181). Among women aged 40–64 years, higher adherence was associated with a higher incidence of low HDL-cholesterolemia, while the prevalence of hypercholesterolemia and HbA1c was lower across higher adherence levels (p for trend = 0.0111; p for trend = 0.0433). Among women aged 65 years or older, higher adherence was consistently associated with a lower incidence of hypertension and impaired FBG and a higher incidence of an elevated TG/HDL ratio (p for trend = 0.0105; 0.0269; 0.0480). The HDL and TG/HDL findings observed for women may reflect the combined influence of multiple factors that can affect HDL and triglyceride metabolism, including hormonal changes related to the menopausal transition and lifestyle factors such as physical activity [36,37]. Therefore, these findings should be interpreted with caution, given the possibility of residual confounding. Furthermore, further studies, including intervention studies that consider these related factors, are warranted.

Recent dietary guidelines in several countries have emphasized the importance of adequate protein intake, including animal-source foods, particularly for older adults and populations at risk of insufficient nutrient intake [38]. In contrast, the Planetary Health Diet primarily emphasizes sustainability and reduced red meat consumption. The mixed lipid marker patterns observed among women in the present study, including HDL-cholesterol and TG/HDL ratio trends, may reflect complex relationships between dietary composition, metabolic markers, and demographic factors such as age and menopausal status. Additionally, the absence of significant associations among men may be related to differences in dietary behaviors, metabolic responses, or lifestyle factors. These findings highlight the importance of interpreting sustainability-oriented dietary patterns within the broader context of current nutritional recommendations and population-specific dietary habits.

Using data from the European Prospective Investigation into Cancer and Nutrition (EPIC)-Oxford cohort, Knuppel et al. examined the associations between major health outcomes, including ischemic heart disease, stroke, diabetes, and all-cause mortality, and the Planetary Health Diet score [19]. Higher EAT-Lancet scores were associated with lower total cholesterol levels, a lower BMI, and lower systolic and diastolic blood pressure, which is consistent with the findings of the present study. However, unlike our results, HDL-C levels increased significantly among individuals with higher adherence. Additionally, although not fully confirmed, compared with the lowest quartile, the highest adherence quartile showed a reduced risk of diabetes and ischemic heart disease.

Previous studies using similar methods for calculating sustainable dietary indices have shown associations with type 2 diabetes and obesity [13,14,35,39]. In a study of a major Danish cohort, there was no strong association between EAT-Lancet scores and body weight over 5 years; however, higher adherence was associated with a reduced risk of obesity and lower increases in waist circumference among individuals with baseline obesity or high waist circumference [13,14]. Studies from Denmark and the United Kingdom also indicated that higher adherence reduced the risk of type 2 diabetes [13,14,39]. In the French NutriNet-Santé cohort, adherence to the Planetary Health Diet, as determined using the EAT-Lancet Diet Index (ELD-I), was associated with cancer incidence but not with cardiovascular disease in selected groups [35].

Studies conducted in Brazil, the United States, Mexico, Sweden, France, and the Netherlands using different sustainable dietary index calculations have also reported associations with type 2 diabetes, obesity, and cardiovascular disease [15–17,22–24,34,35]. For example, studies of Mexican female teachers and of Swedish cohorts revealed that higher adherence reduced the risk of type 2 diabetes [15,22]. Sustainable diet indices developed in Brazil and France have revealed an association between a sustainable diet andlower obesity risk [16,23]. In the ELSA-Brasil cohort, higher adherence to the Planetary Health Diet, as determined using the Planetary Health Diet Index (PHDI), was linked to improvements in obesity-related indicators [23], and in the French NutriNet-Santé cohort, adherence to a sustainable diet, as determined using the Sustainable Diet Index (SDI) was associated with the prevention of weight gain and a reduced risk of overweight and obesity [16].

In a study of the EPIC-NL cohort in the Netherlands, higher adherence to the Healthy Reference Diet (HRD), a sustainability-oriented diet, was associated with a lower risk of heart disease and coronary heart disease [24]. In contrast, findings from the ELSA-Brasil cohort revealed that higher adherence to the Planetary Health Diet, as determined using the Planetary Health Diet Index (PHDI), was associated with lower blood pressure and reduced levels of total cholesterol, LDL-cholesterol, and non-HDL-cholesterol [34]. Analyses of data from the National Health and Nutrition Examination Survey (NHANES 2015–2018) revealed that sustainable dietary indices were not significantly associated with FBG but were linked to lower TG levels [40,41]. Moreover, a German study revealed no association between the Dietary Index (DI), a sustainability-focused index, and cardiometabolic risk indicators [17].

This study has several limitations. First, because the analysis was based on cross-sectional data from the National Health and Nutrition Examination Survey, causal relationships between Planetary Health Diet scores and the metabolic risk factors examined in this study cannot be inferred. The cross-sectional design of this study precludes the inference of causal relationships; thus, the possibility of reverse causation cannot be ruled out, as individuals with existing metabolic risks may have altered their diets prior to the survey. Second, dietary intake was assessed using 24-hour recall, which relies on participant memory and may not fully represent habitual dietary patterns. Since this assessment relies on a single 24-hour recall, it may not fully reflect long-term habitual patterns or capture day-to-day variability in nutrient intake. Third, the study examined associations with intermediate metabolic indicators rather than diagnosed disease outcomes, limiting comparisons with previous research conducted on chronic disease incidence. Furthermore, in this study, the TG/HDL ratio was utilized as a cardiometabolic risk marker; future studies should incorporate biomarker-based measures to more accurately validate the sex-specific findings of the present study.

Nevertheless, this study has important strengths. By analyzing men and women separately, sex-specific associations could be identified. Additionally, unlike previous studies, this analysis examined associations across detailed age groups, providing a clearer understanding of how dietary patterns aligned with the Planetary Health Diet are associated with metabolic health indicators throughout adulthood.

Despite the limitations, this study contributes meaningful evidence regarding the EAT-Lancet dietary recommendations, for which research in Korea remains limited. The findings highlight associations between dietary patterns aligned with the Planetary Health Diet and several metabolic risk factors that are key predictors of noncommunicable and chronic diseases.

## Сonclusion

In conclusion, higher Planetary Health Diet scores, reflecting dietary patterns on the day of dietary recall, were associated with selected cardiometabolic markers among Korean women, whereas corresponding associations were not clearly observed among men. These findings should be interpreted cautiously because the cross-sectional design of this study does not permit causal inference, and dietary intake was assessed using a single 24-hour recall, which may not adequately reflect usual long-term intake. Future studies using longitudinal dietary assessments and biomarker-based measures of cardiometabolic risk are needed to confirm the findings and clarify the observed sex-specific associations in this study.

## Supporting information

S1 Table Components, scoring criteria, and food item mapping for the Planetary Health Diet score. (PDF)

S2 Table Least-squares means (95% confidence intervals) of health outcomes across quartiles of the Planetary Health Diet Score by age group (19–39, 40–64, and ≥65 years) and sex. (PDF)

S3 Table Odds ratios (95% confidence intervals) for health outcomes across quartiles of the Planetary Health Diet Score by age group (19–39, 40–64, and ≥65 years) and sex. (PDF)

## References

[1] Budreviciute A, Damiati S, Sabir DK, Onder K, Schuller-Goetzburg P, Plakys G, et al. Management and Prevention Strategies for Non-communicable Diseases (NCDs) and their risk factors. Front Public Health. 2020;8:574111. doi: 10.3389/fpubh.2020.574111 33324597 PMC7726193

[2] World Health Organization. Climate change and noncommunicable diseases: connections 2023. https://www.who.int/news/item/02-11-2023-climate-change-and-noncommunicable-diseases-connections

[3] World Health Organization. Health at a glance | Republic of Korea 2024. https://srhdpeuwpubsa.blob.core.windows.net/whdh/DATADOT/COUNTRY/PDF/410_Republic%20of%20Korea.pdf

[4] Lee M-J, Popkin BM, Kim S. The unique aspects of the nutrition transition in South Korea: The retention of healthful elements in their traditional diet. Public Health Nutr. 2002;5(1A):197–203. doi: 10.1079/PHN2001294 12027285

[5] Kim S, Moon S, Popkin BM. The nutrition transition in South Korea. Am J Clin Nutr. 2000;71(1):44–53. doi: 10.1093/ajcn/71.1.44 10617945

[6] Woo HD, Shin A, Kim J. Dietary patterns of Korean adults and the prevalence of metabolic syndrome: A cross-sectional study. PLoS One. 2014;9(11):e111593. doi: 10.1371/journal.pone.0111593 25365577 PMC4218781

[7] Malik VS, Popkin BM, Bray GA, Després J-P, Hu FB. Sugar-sweetened beverages, obesity, type 2 diabetes mellitus, and cardiovascular disease risk. Circulation. 2010;121(11):1356–64. doi: 10.1161/CIRCULATIONAHA.109.876185 20308626 PMC2862465

[8] Bechthold A, Boeing H, Schwedhelm C, Hoffmann G, Knüppel S, Iqbal K, et al. Food groups and risk of coronary heart disease, stroke and heart failure: A systematic review and dose-response meta-analysis of prospective studies. Crit Rev Food Sci Nutr. 2019;59(7):1071–90. doi: 10.1080/10408398.2017.1392288 29039970

[9] Larsson SC, Wolk A. Meat consumption and risk of colorectal cancer: A meta-analysis of prospective studies. Int J Cancer. 2006;119(11):2657–64. doi: 10.1002/ijc.22170 16991129

[10] Ioniță-Mîndrican C-B, Ziani K, Mititelu M, Oprea E, Neacșu SM, Moroșan E, et al. Therapeutic benefits and dietary restrictions of fiber intake: A state of the art review. Nutrients. 2022;14(13):2641. doi: 10.3390/nu14132641 35807822 PMC9268622

[11] Burlingame B, Dernini S. Sustainable diets and biodiversity: Directions and solutions for policy, research and action. Burlingame B, Dernini S. Food and Agriculture Organization of the United Nations (FAO). 2012.

[12] Willett W, Rockström J, Loken B, Springmann M, Lang T, Vermeulen S, et al. Food in the Anthropocene: The EAT-Lancet Commission on healthy diets from sustainable food systems. Lancet. 2019;393(10170):447–92. doi: 10.1016/S0140-6736(18)31788-4 30660336

[13] Langmann F, Ibsen DB, Tjønneland A, Olsen A, Overvad K, Dahm CC. Adherence to the EAT-Lancet diet in midlife and development in weight or waist circumference after five years in a Danish cohort. Dialogues Health. 2023;3:100151. doi: 10.1016/j.dialog.2023.100151 38515808 PMC10953849

[14] Langmann F, Ibsen DB, Tjønneland A, Olsen A, Overvad K, Dahm CC. Adherence to the EAT-Lancet diet is associated with a lower risk of type 2 diabetes: The Danish Diet, Cancer and Health cohort. Eur J Nutr. 2023;62(3):1493–502. doi: 10.1007/s00394-023-03090-3 36688993

[15] Zhang S, Stubbendorff A, Olsson K, Ericson U, Niu K, Qi L, et al. Adherence to the EAT-Lancet diet, genetic susceptibility, and risk of type 2 diabetes in Swedish adults. Metabolism. 2023;141:155401. doi: 10.1016/j.metabol.2023.155401 36682448

[16] Seconda L, Egnell M, Julia C, Touvier M, Hercberg S, Pointereau P, et al. Association between sustainable dietary patterns and body weight, overweight, and obesity risk in the NutriNet-Santé prospective cohort. Am J Clin Nutr. 2020;112(1):138–49. doi: 10.1093/ajcn/nqz259 31725157

[17] Montejano Vallejo R, Schulz C-A, van de Locht K, Oluwagbemigun K, Alexy U, Nöthlings U. Associations of Adherence to a Dietary Index Based on the EAT-Lancet Reference Diet with Nutritional, Anthropometric, and Ecological Sustainability Parameters: Results from the German DONALD Cohort Study. J Nutr. 2022;152(7):1763–72. doi: 10.1093/jn/nxac094 35554563 PMC9258554

[18] Martins LB, Gamba M, Stubbendorff A, Gasser N, Löbl L, Stern F, et al. Association between the EAT-Lancet Diet, Incidence of Cardiovascular Events, and All-Cause Mortality: Results from a Swiss Cohort. J Nutr. 2025;155(2):483–91. doi: 10.1016/j.tjnut.2024.12.012 39742968

[19] Knuppel A, Papier K, Key TJ, Travis RC. EAT-Lancet score and major health outcomes: The EPIC-Oxford study. Lancet. 2019;394(10194):213–4. doi: 10.1016/S0140-6736(19)31236-X 31235280

[20] Lee U, Kim S, Kang E, Lee H, Lee S, Lee JE, et al. Sociodemographic and lifestyle factors associated with the Planetary Health diet in the Korean adult population. PLoS One. 2026;21(4):e0345467. doi: 10.1371/journal.pone.0345467 41984757 PMC13082601

[21] Lin X, Wang S, Huang J. The Association between the EAT-Lancet Diet and Diabetes: A systematic review. Nutrients. 2023;15(20):4462. doi: 10.3390/nu15204462 37892537 PMC10610026

[22] López GE, Batis C, González C, Chávez M, Cortés-Valencia A, López-Ridaura R, et al. EAT-Lancet Healthy Reference Diet score and diabetes incidence in a cohort of Mexican women. Eur J Clin Nutr. 2023;77(3):348–55. doi: 10.1038/s41430-022-01246-8 36471166

[23] Cacau LT, Benseñor IM, Goulart AC, Cardoso LO, Lotufo PA, Moreno LA, et al. Adherence to the planetary health diet index and obesity indicators in the brazilian longitudinal study of adult health (ELSA-Brasil). Nutrients. 2021;13(11):3691. doi: 10.3390/nu13113691 34835947 PMC8625681

[24] Colizzi C, Harbers MC, Vellinga RE, Verschuren WMM, Boer JMA, Biesbroek S, et al. Adherence to the EAT-Lancet healthy reference diet in relation to risk of cardiovascular events and environmental impact: Results From the EPIC-NL Cohort. J Am Heart Assoc. 2023;12(8):e026318. doi: 10.1161/JAHA.122.026318 37066787 PMC10227249

[25] Choi SA, Chung SS, Rho JO. Benefits of adherence to the Korea Healthy Eating Index on the risk factors and incidence of the metabolic syndrome: Analysis of the 7th (2016–2018) Korea National Health and Nutrition Examination Survey. J Nutr Health. 2022;55(1):120. doi: 10.4163/jnh.2022.55.1.120

[26] Deierlein AL, Morland KB, Scanlin K, Wong S, Spark A. Diet quality of urban older adults age 60 to 99 years: The Cardiovascular Health of Seniors and Built Environment Study. J Acad Nutr Diet. 2014;114(2):279–87. doi: 10.1016/j.jand.2013.09.002 24262516 PMC3946974

[27] Grech A, Sui Z, Siu HY, Zheng M, Allman-Farinelli M, Rangan A. Socio-demographic determinants of diet quality in australian adults using the validated healthy eating index for Australian Adults (HEIFA-2013). Healthcare (Basel). 2017;5(1):7. doi: 10.3390/healthcare5010007 28165394 PMC5371913

[28] McCullough ML, Feskanich D, Stampfer MJ, Rosner BA, Hu FB, Hunter DJ, et al. Adherence to the Dietary Guidelines for Americans and risk of major chronic disease in women. Am J Clin Nutr. 2000;72(5):1214–22. doi: 10.1093/ajcn/72.5.1214 11063452

[29] Kang M, Park S-Y, Shvetsov YB, Wilkens LR, Marchand LL, Boushey CJ, et al. Sex differences in sociodemographic and lifestyle factors associated with diet quality in a multiethnic population. Nutrition. 2019;66:147–52. doi: 10.1016/j.nut.2018.11.022 31288218 PMC7085987

[30] Andrews RR, Anderson KR, Fry JL. Sex-specific variation in metabolic responses to diet. Nutrients. 2024;16(17):2921. doi: 10.3390/nu16172921 39275236 PMC11397081

[31] Yi Y, An J. Sex differences in risk factors for metabolic syndrome in the Korean population. Int J Environ Res Public Health. 2020;17(24):9513. doi: 10.3390/ijerph17249513 33353082 PMC7766635

[32] Mun J, Kim S, Kim K, Nam C, Kim S, Park CY. Accuracy of 24-hour dietary recalls among free-living older korean adults: Validation against weighed intakes. J Nutr. 2025;155(12):4446–54. doi: 10.1016/j.tjnut.2025.10.038 41173375

[33] Jung S. Sustainable diets: A scoping review and descriptive study of concept, measurement, and suggested methods for the development of Korean version. Korean J Community Nutr. 2024;29(1):34–50. doi: 10.5720/kjcn.2024.29.1.34 41473128 PMC12746556

[34] Cacau LT, Benseñor IM, Goulart AC, Cardoso L de O, Santos I de S, Lotufo PA, et al. Adherence to the EAT-Lancet sustainable reference diet and cardiometabolic risk profile: cross-sectional results from the ELSA-Brasil cohort study. Eur J Nutr. 2023;62(2):807–17. doi: 10.1007/s00394-022-03032-5 36266476

[35] Berthy F, Brunin J, Allès B, Fezeu LK, Touvier M, Hercberg S, et al. Association between adherence to the EAT-Lancet diet and risk of cancer and cardiovascular outcomes in the prospective NutriNet-Santé cohort. Am J Clin Nutr. 2022;116(4):980–91. doi: 10.1093/ajcn/nqac208 35918246

[36] El Khoudary SR, Chen X, Nasr AN, Billheimer J, Brooks MM, McConnell D. HDL (High-Density Lipoprotein) subclasses, lipid content, and function trajectories across the menopause transition: SWAN-HDL Study. Arteriosclerosis, Thrombosis, and Vascular Biology. 2021;41(2):951–61. doi: 10.1161/atvbaha.120.315355 33267661 PMC8105263

[37] Franczyk B, Gluba-Brzózka A, Ciałkowska-Rysz A, Ławiński J, Rysz J. The Impact of Aerobic Exercise on HDL Quantity and Quality: A narrative review. Int J Mol Sci. 2023;24(5):4653. doi: 10.3390/ijms24054653 36902082 PMC10003711

[38] Sheffield S, Fiorotto ML, Davis TA. Nutritional importance of animal-sourced foods in a healthy diet. Front Nutr. 2024;11:1424912. doi: 10.3389/fnut.2024.1424912 39119462 PMC11306033

[39] Xu C, Cao Z, Yang H, Hou Y, Wang X, Wang Y. Association Between the EAT-lancet diet pattern and risk of type 2 diabetes: A prospective cohort study. Front Nutr. 2022;8:784018. doi: 10.3389/fnut.2021.784018 35096931 PMC8795697

[40] Frank SM, Jaacks LM, Adair LS, Avery CL, Meyer K, Rose D, et al. Adherence to the Planetary Health Diet Index and correlation with nutrients of public health concern: An analysis of NHANES 2003-2018. Am J Clin Nutr. 2024;119(2):384–92. doi: 10.1016/j.ajcnut.2023.10.018 38309827 PMC10884610

[41] Frank SM, Jaacks LM, Avery CL, Adair LS, Meyer K, Rose D. Dietary quality and cardiometabolic indicators in the USA: A comparison of the Planetary Health Diet Index, Healthy Eating Index-2015, and Dietary Approaches to Stop Hypertension. PLoS One. 2024;19(1):e0296069. doi: 10.1371/journal.pone.0296069 PMC10781024 38198440

