# Supplementary material for: Association of the planetary health diet score with obesity, high blood pressure, dyslipidemia, and cardiometabolic risk markers: Using data from the 2016–2020 Korea National Health and Nutrition Examination Survey
Source: PLoS One. 2026 Jun 8;21(6):e0350821. doi: 10.1371/journal.pone.0350821 (PMC13245749; doi:10.1371/journal.pone.0350821)
Supplement: S1 Table — (PDF) [file pone.0350821.s001.pdf]

Table A. Scoring criteria and food item mapping for the EAT-Lancet diet score

Dietary components	Criteria for scoring 1 point*	Food items included in the component
Whole grains	≤464 g/day and whole grain fiber >5 g	Whole- and refined-grain foods, including rice, oats, wheat, rye, barley, sorghum, corn, brown rice, millet, quinoa, Job's tears, cereals, breads, crackers, cakes, rice cakes, porridge, noodles, and other grain- or flour-based foods.
Potatoes and cassava	≤100 g/day	Potato, potato chips/snacks, French fried, hashed brown
all vegetables	≥200 g/day	Vegetables, including non-salted vegetables and kimchi/salted vegetables, such as sweet potato, yam, taro, eggplant, leafy greens, peppers, cauliflower, carrot, radish, Chinese cabbage, broccoli, lettuce, spinach, cabbage, onion, cucumber, tomato, pumpkin, mushrooms, herbs, and kimchi or pickled vegetables.
all fruits	≥100 g/day	Fruits, including coconut, persimmon, citrus fruits, strawberry, mango, melon, banana, pear, cherry, peach, blueberry, plum, grape, watermelon, kiwi, pineapple, papaya, avocado, and other fresh fruits.
whole milk or equivalents	≤500 g/day	Milk, low-fat milk, ice cream, yoghurt, cheese, cream, shake, soups, coffee creamer (milk fat)
beef, lamb, pork	≤28 g/day	Red and processed meats, including pork, beef, lamb, goat meat, meat by-products, bacon, sausage, ham, pressed ham, and beef jerky.
chicken, other poultry	≤58 g/day	Chicken, chicken by-products, duck, turkey, pheasant
Eggs	≤25 g/day	Eggs, quail eggs, duck eggs
Fish	≤100 g/day	Fish and seafood, including tuna, mackerel, flounder, cod, sea bass, anchovy, pollack, croaker, salmon, trout, eel, shellfish, crab, squid, octopus, shrimp, and other marine products.
dry beans, lentils	≤100 g/day	Kidney beans, mung beans, brown soybeans, cowpeas, peas, horse beans, broad beans, red beans
soy foods	≤50 g/day	soybeans tofu natto
peanuts or tree nuts	≥25 g/day	Nuts and seeds, including peanuts, tree nuts, sesame seeds, sunflower seeds, and ginkgo nuts.
palm oil, unsaturated oils, dairy fats including milk, lard or tallow	with a ratio of 0.8 for unsaturated to saturated fat intake	Plant and animal fats/oils, including cooking oil, vegetable-fat coffee creamer, chicken fat, lard, margarine, butter, and beef tallow.
all sweeteners	≤31 g/day	Sweetened foods and beverages, including baked goods, confectionery, cereals, sugar and syrups, sweetened dairy products, sweetened soy milk, fruit/vegetable drinks, soft drinks, tea, coffee, soups, and sauces.

*Each dietary component contributed 0 or 1 point resulting in a total score ranging from 0-14 points.
