# Supplementary material for: Association of the planetary health diet score with obesity, high blood pressure, dyslipidemia, and cardiometabolic risk markers: Using data from the 2016–2020 Korea National Health and Nutrition Examination Survey
Source: PLoS One. 2026 Jun 8;21(6):e0350821. doi: 10.1371/journal.pone.0350821 (PMC13245749; doi:10.1371/journal.pone.0350821)
Supplement: S2 Table — (PDF) [file pone.0350821.s002.pdf]

Table A. Least-squares means (95% confidence intervals) of health outcome according to the Quartiles of EAT-Lancet diet score in men aged 19~39

**LS-means (95% CIs)**	**Quartiles of the EAT-Lancet diet score**				
	**Q1 (n=1,435)**	**Q2 (n=983)**	**Q3 (n=674)**	**Q4 (n=314)**	***p* for trend**
**Body mass index (kg/m²)**					
model 1	26.05 (25.64-26.46)	26.28 (25.85-26.72)	26.11 (25.65-26.57)	25.93 (25.37-26.49)	0.9173
model 2	26.00 (25.44-26.56)	26.22 (25.66-26.78)	26.06 (25.50-26.62)	25.87 (25.21-26.53)	0.8854
**Waist circumferences (cm)**					
model 1	91.44 (90.38-92.50)	91.78 (90.65-92.92)	91.37 (90.18-92.55)	91.22 (89.72-92.72)	0.7863
model 2	91.10 (89.66-92.54)	91.44 (89.97-92.92)	91.04 (89.57-92.51)	90.90 (89.15-92.64)	0.8031
**Systolic blood pressure (mmHg)**					
model 1	118.57 (117.35-119.78)	118.66 (117.36-119.95)	118.74 (117.29-120.18)	118.07 (116.27-119.86)	0.7901
model 2	119.49 (117.90-121.09)	119.62 (117.91-121.33)	119.62 (117.88-121.36)	118.98 (116.91-121.05)	0.7539
**Diastolic blood pressure (mmHg)**					
model 1	83.87 (82.81-84.93)	83.67 (82.59-84.75)	83.75 (82.58-84.92)	83.47 (82.10-84.85)	0.5563
model 2	84.68 (83.32-86.03)	84.50 (83.09-85.92)	84.51 (83.09-85.92)	84.26 (82.68-85.84)	0.5022
**Triglyceride (mg/dL)**					
model 1	209.60 (194.10-225.10)	203.64 (190.46-216.83)	206.28 (191.67-220.89)	213.78 (190.97-236.59)	0.9901
model 2	204.55 (183.57-225.53)	199.59 (180.72-218.46)	202.27 (182.93-221.60)	210.22 (184.38-236.07)	0.8173
**Hight-density lipoprotein (mg/dL)**					
model 1	46.16 (44.94-47.37)	46.68 (45.47-47.90)	46.66 (45.27-48.05)	46.65 (45.03-48.27)	0.3400
model 2	46.46 (44.89-48.03)	46.96 (45.43-48.48)	46.92 (45.25-48.59)	47.03 (45.16-48.90)	0.3321
**Total cholesterol (mg/dL)**					
model 1	218.97 (215.39-222.54)	219.14 (215.45-222.82)	219.28 (215.37-223.19)	219.97 (214.91-225.03)	0.6881
model 2	216.55 (211.82-221.28)	216.77 (211.89-221.65)	216.97 (212.07-221.86)	217.75 (211.95-223.55)	0.6186
**TG/HDL**					
model 1	5.23 (4.72-5.74)	4.99 (4.56-5.41)	5.09 (4.62-5.55)	5.52 (4.61-6.43)	0.7809
model 2	5.03 (4.41-5.65)	4.81 (4.28-5.35)	4.92 (4.36-5.47)	5.35 (4.42-6.28)	0.6741
**Fasting blood glucose (mg/dL)**					
model 1	102.38 (100.85-103.91)	102.85 (101.23-104.48)	102.56 (100.48-104.64)	102.73 (100.55-104.91)	0.6732
model 2	103.19 (100.63-105.75)	103.78 (100.98-106.59)	103.39 (100.07-106.72)	103.58 (100.56-106.61)	0.6271
**HbA1c (%)**					
model 1	5.65 (5.60-5.69)	5.68 (5.62-5.73)	5.66 (5.59-5.73)	5.62 (5.56-5.68)	0.8156
model 2	5.70 (5.63-5.77)	5.73 (5.66-5.81)	5.71 (5.62-5.80)	5.67 (5.60-5.75)	0.7438

Abbreviations: LS-means, least-squares means; CIs, confidence intervals; Q, quartile; TG/HDL, triglyceride to HDL-cholesterol ratio: HbA1c, hemoglobin A_{1C}.
model 1 adjusted for age (year, continuous) and energy intake (kcal, continuous)
model 2 additionally adjusted for household income (low, middle-low, middle-high, high), smoking status (never, past, ever), marriage status (married, unmarried), education level (elementary school or below, middle school, high school, college or above), prevalence of monthly alcohol use(No, Yes), physical activity (No, Yes)

Table B. Least-squares means (95% confidence intervals) of health outcome according to the Quartiles of EAT-Lancet diet score in men aged 40~64

	Quartiles of the EAT-Lancet diet score				
	**Q1**	**Q2**	**Q3**	**Q4**	***p* for trend**
**LS-means (95% CIs)**	**(n=1,270)**	**(n=1,306)**	**(n=1,327)**	**(n=962)**	
**Body mass index (kg/m²)**					
model 1	25.05 (24.83-25.27)	25.04 (24.82-25.27)	25.08 (24.85-25.31)	24.85 (24.60-25.10)	0.3241
model 2	25.04 (24.78-25.30)	25.02 (24.76-25.28)	25.03 (24.76-25.30)	24.79 (24.50-25.07)	0.1712
**Waist circumferences (cm)**					
model 1	87.90 (87.32-88.49)	87.60 (87.00-88.19)	87.62 (86.99-88.25)	86.98 (86.27-87.69)	0.0523
model 2	87.55 (86.85-88.25)	87.24 (86.55-87.94)	87.19 (86.45-87.94)	86.54 (85.73-87.35)	0.0281
**Systolic blood pressure (mmHg)**					
model 1	118.23 (117.28-119.18)	119.99 (118.99-120.98)	119.26 (118.25-120.27)	118.52 (117.46-119.59)	0.7479
model 2	118.58 (117.52-119.64)	120.23 (119.11-121.35)	119.36 (118.28-120.44)	118.77 (117.62-119.93)	0.9326
**Diastolic blood pressure (mmHg)**					
model 1	81.04 (80.39-81.69)	81.93 (81.23-82.62)	81.29 (80.57-82.02)	81.29 (80.51-82.07)	0.8824
model 2	81.01 (80.26-81.76)	81.81 (81.01-82.61)	81.09 (80.30-81.89)	81.19 (80.34-82.04)	0.9376
**Triglyceride (mg/dL)**					
model 1	190.23 (180.52-199.95)	187.62 (177.41-197.82)	193.96 (183.25-204.68)	195.58 (182.04-209.13)	0.3605
model 2	180.80 (170.14-191.45)	178.98 (167.45-190.51)	184.71 (173.41-196.00)	188.80 (173.91-203.70)	0.2282
**Hight-density lipoprotein (mg/dL)**					
model 1	46.42 (45.70-47.14)	47.31 (46.55-48.07)	47.28 (46.53-48.02)	46.27 (45.39-47.15)	0.9854
model 2	46.50 (45.69-47.30)	47.20 (46.37-48.02)	47.13 (46.34-47.92)	46.25 (45.32-47.18)	0.8383
**Total cholesterol (mg/dL)**					
model 1	204.25 (202.03-206.47)	205.14 (202.70-207.59)	203.63 (201.21-206.04)	203.81 (200.80-206.83)	0.5965
model 2	202.41 (199.85-204.98)	203.26 (200.54-205.97)	201.71 (199.07-204.36)	201.99 (198.89-205.09)	0.5960
**TG/HDL**					
model 1	4.61 (4.31-4.90)	4.54 (4.19-4.88)	4.66 (4.32-5.01)	4.87 (4.44-5.30)	0.2685
model 2	4.37 (4.03-4.70)	4.34 (3.93-4.74)	4.45 (4.09-4.81)	4.71 (4.24-5.18)	0.1607
**Fasting blood glucose (mg/dL)**					
model 1	101.30 (99.87-102.73)	101.35 (100.11-102.59)	101.93 (100.55-103.31)	102.34 (100.51-104.16)	0.2805
model 2	101.63 (99.99-103.26)	101.67 (100.19-103.15)	102.12 (100.41-103.82)	102.58 (100.67-104.48)	0.3407
**HbA1c (%)**					
model 1	5.67 (5.62-5.72)	5.64 (5.60-5.69)	5.63 (5.58-5.67)	5.66 (5.61-5.72)	0.6610
model 2	5.66 (5.60-5.71)	5.64 (5.59-5.69)	5.62 (5.57-5.67)	5.66 (5.59-5.72)	0.7024

Abbreviations: LS-means, least-squares means; CIs, confidence intervals; Q, quartile; TG/HDL, triglyceride to HDL-cholesterol ratio: HbA1c, hemoglobin A_{1C}.
model 1 adjusted for age (year, continuous) and energy intake (kcal, continuous)
model 2 additionally adjusted for household income (low, middle-low, middle-high, high), smoking status (never, past, ever), marriage status (married, unmarried), education level (elementary school or below, middle school, high school, college or above), prevalence of monthly alcohol use(No, Yes), physical activity (No, Yes)

Table C. Least-squares means (95% confidence intervals) of health outcome according to the Quartiles of EAT-Lancet diet score in men aged 65 and above

LS-means (95% CIs)	Quartiles of the EAT-Lancet diet score Q1 (n=244)	Q2 (n=534)	Q3 (n=702)	Q4 (n=879)	*p* for trend
**Body mass index (kg/m²)**					
model 1	26.13 (25.27-26.98)	25.62 (24.84-26.39)	25.99 (25.20-26.77)	26.20 (25.44-26.96)	0.0301
model 2	25.74 (24.78-26.71)	25.20 (24.31-26.09)	25.54 (24.65-26.44)	25.75 (24.88-26.61)	0.0617
**Waist circumferences (cm)**					
model 1	89.25 (86.54-91.97)	88.14 (85.66-90.62)	88.68 (86.19-91.16)	89.26 (86.85-91.68)	0.2485
model 2	87.67 (84.70-90.65)	86.53 (83.76-89.29)	87.00 (84.23-89.76)	87.60 (84.89-90.31)	0.3005
**Systolic blood pressure (mmHg)**					
model 1	118.17 (113.18-123.16)	116.75 (112.20-121.31)	117.21 (112.60-121.82)	116.95 (112.51-121.39)	0.6144
model 2	117.35 (110.94-123.75)	115.88 (110.14-121.62)	116.17 (110.28-122.06)	115.87 (110.12-121.62)	0.4544
**Diastolic blood pressure (mmHg)**					
model 1	87.10 (84.21-89.99)	87.62 (84.95-90.29)	87.35 (84.71-90.00)	87.91 (85.28-90.54)	0.3194
model 2	87.10 (83.55-90.65)	87.53 (84.29-90.76)	87.20 (83.95-90.45)	87.76 (84.54-90.98)	0.4201
**Triglyceride (mg/dL)**					
model 1	180.96 (158.32-203.59)	186.48 (164.37-208.58)	190.63 (164.89-216.38)	182.99 (161.12-204.86)	0.8844
model 2	177.78 (152.73-202.83)	183.86 (158.48-209.23)	188.27 (160.13-216.40)	181.62 (156.70-206.54)	0.8251
**Hight-density lipoprotein (mg/dL)**					
model 1	47.88 (44.63-51.12)	48.58 (45.54-51.62)	48.35 (45.25-51.46)	47.55 (44.47-50.63)	0.2741
model 2	46.91 (43.39-50.42)	47.38 (44.04-50.71)	47.09 (43.69-50.48)	46.41 (43.08-49.74)	0.2300
**Total cholesterol (mg/dL)**					
model 1	201.88 (190.79-212.96)	205.44 (195.62-215.27)	201.00 (191.13-210.86)	200.79 (190.87-210.70)	0.1500
model 2	201.71 (189.27-214.15)	205.23 (193.72-216.75)	200.67 (189.22-212.12)	200.51 (189.08-211.94)	0.1386
**TG/HDL**					
model 1	4.27 (3.59-4.95)	4.39 (3.71-5.07)	4.53 (3.71-5.35)	4.38 (3.71-5.06)	0.6254
model 2	4.26 (3.49-5.02)	4.41 (3.63-5.19)	4.56 (3.66-5.46)	4.43 (3.66-5.20)	0.4038
**Fasting blood glucose (mg/dL)**					
model 1	105.66 (101.03-110.28)	105.86 (101.21-110.51)	106.36 (101.51-111.21)	106.05 (101.15-110.94)	0.7365
model 2	105.57 (100.43-110.72)	105.72 (100.35-111.09)	106.19 (100.79-111.60)	105.98 (100.47-111.48)	0.6954
**HbA1c (%)**					
model 1	5.66 (5.51-5.81)	5.67 (5.52-5.82)	5.72 (5.57-5.87)	5.66 (5.51-5.82)	0.9436
model 2	5.64 (5.47-5.80)	5.65 (5.48-5.81)	5.70 (5.54-5.87)	5.64 (5.48-5.81)	0.9068

Abbreviations: LS-means, least-squares means; CIs, confidence intervals; Q, quartile; TG/HDL, triglyceride to HDL-cholesterol ratio: HbA1c, hemoglobin A_{1C}.
model 1 adjusted for age (year, continuous) and energy intake (kcal, continuous)
model 2 additionally adjusted for household income (low, middle-low, middle-high, high), smoking status (never, past, ever), marriage status (married, unmarried), education level (elementary school or below, middle school, high school, college or above), prevalence of monthly alcohol use(No, Yes), physical activity (No, Yes)

Table D. Least-squares means (95% confidence intervals) of health outcome according to the Quartiles of EAT-Lancet diet score in women aged 19~39

	**Quartiles of the EAT-Lancet diet score**				
**LS-means (95% CIs)**	**Q1 (n=1,375)**	**Q2 (n=1,259)**	**Q3 (n=1,011)**	**Q4 (n=522)**	**p for trend**
**Body mass index (kg/m²)**					
model 1	23.58 (23.12-24.04)	23.32 (22.89-23.75)	23.11 (22.68-23.55)	23.18 (22.65-23.71)	0.0236
model 2	23.70 (22.87-24.52)	23.40 (22.59-24.21)	23.24 (22.39-24.09)	23.28 (22.40-24.15)	0.0194
**Waist circumferences (cm)**					
model 1	80.53 (79.40-81.67)	79.71 (78.62-80.79)	79.46 (78.38-80.54)	79.43 (78.10-80.76)	0.0184
model 2	81.19 (79.00-83.39)	80.26 (78.08-82.43)	80.11 (77.86-82.35)	80.06 (77.75-82.38)	0.0155
**Systolic blood pressure (mmHg)**					
model 1	107.51 (106.30-108.72)	106.64 (105.39-107.90)	107.06 (105.81-108.32)	107.33 (106.03-108.64)	0.6044
model 2	107.76 (105.11-110.41)	106.90 (104.17-109.64)	107.37 (104.63-110.12)	107.64 (104.91-110.37)	0.6913
**Diastolic blood pressure (mmHg)**					
model 1	73.57 (72.61-74.54)	72.56 (71.59-73.53)	73.12 (72.17-74.07)	72.81 (71.77-73.86)	0.1246
model 2	73.66 (71.21-76.11)	72.69 (70.21-75.16)	73.25 (70.80-75.69)	72.97 (70.47-75.47)	0.1654
**Triglyceride (mg/dL)**					
model 1	107.43 (101.21-113.65)	106.11 (98.97-113.26)	105.11 (98.70-111.51)	102.74 (95.88-109.60)	0.1150
model 2	133.07 (105.93-160.21)	131.75 (104.35-159.15)	130.90 (103.93-157.87)	128.52 (101.68-155.37)	0.1247
**Hight-density lipoprotein (mg/dL)**					
model 1	57.27 (55.86-58.68)	57.60 (56.31-58.89)	58.19 (56.76-59.61)	57.07 (55.49-58.66)	0.5456
model 2	57.93 (54.46-61.40)	58.63 (55.19-62.08)	59.00 (55.51-62.50)	58.05 (54.52-61.59)	0.2826
**Total cholesterol (mg/dL)**					
model 1	198.03 (194.43-201.63)	197.91 (194.56-201.26)	197.84 (194.16-201.52)	198.79 (194.28-203.30)	0.8162
model 2	207.00 (199.59-214.41)	207.05 (199.83-214.26)	206.94 (199.46-214.42)	207.79 (199.78-215.80)	0.7828
**TG/HDL**					
model 1	2.12 (1.95-2.28)	2.10 (1.89-2.32)	2.06 (1.89-2.23)	2.03 (1.84-2.22)	0.2055
model 2	2.68 (2.01-3.34)	2.65 (1.96-3.34)	2.62 (1.95-3.28)	2.58 (1.92-3.24)	0.1660
**Fasting blood glucose (mg/dL)**					
model 1	95.47 (94.20-96.73)	95.38 (93.93-96.84)	95.57 (93.95-97.20)	94.92 (93.37-96.46)	0.6215
model 2	95.95 (92.97-98.93)	95.88 (92.91-98.85)	96.09 (92.96-99.23)	95.49 (92.46-98.52)	0.7193
**HbA1c (%)**					
model 1	5.47 (5.43-5.51)	5.46 (5.41-5.51)	5.45 (5.40-5.50)	5.44 (5.39-5.49)	0.1115
model 2	5.59 (5.50-5.69)	5.58 (5.48-5.68)	5.57 (5.46-5.68)	5.56 (5.46-5.66)	0.0736

Abbreviations: LS-means, least-squares means; CIs, confidence intervals; Q, quartile; TG/HDL, triglyceride to HDL-cholesterol ratio: HbA1c, hemoglobin A1C.
model 1 adjusted for age (year, continuous) and energy intake (kcal, continuous)
model 2 additionally adjusted for household income (low, middle-low, middle-high, high), smoking status (never, past, ever), marriage status (married, unmarried), education level (elementary school or below, middle school, high school, college or above), prevalence of monthly alcohol use(No, Yes), physical activity (No, Yes), menopause status (No, Yes)

Table E. Least-squares means (95% confidence intervals) of health outcome according to the Quartiles of EAT-Lancet diet score in women aged 40~64

	Quartiles of the EAT-Lancet diet score				
	**Q1**	**Q2**	**Q3**	**Q4**	**p for trend**
**LS-means (95% CIs)**	**(n=1,268)**	**(n=1,813)**	**(n=2,137)**	**(n=2,046)**	
**Body mass index (kg/m²)**					
model 1	23.23 (22.99-23.47)	23.28 (23.07-23.48)	23.35 (23.16-23.54)	23.22 (23.02-23.42)	0.9859
model 2	23.37 (23.11-23.62)	23.37 (23.15-23.59)	23.44 (23.23-23.65)	23.30 (23.08-23.52)	0.7311
**Waist circumferences (cm)**					
model 1	77.97 (77.36-78.58)	78.34 (77.80-78.88)	78.46 (77.95-78.96)	78.00 (77.48-78.51)	0.9848
model 2	78.29 (77.62-78.96)	78.50 (77.92-79.08)	78.64 (78.07-79.21)	78.18 (77.60-78.76)	0.7684
**Systolic blood pressure (mmHg)**					
model 1	112.43 (111.52-113.34)	113.16 (112.31-114.02)	113.11 (112.33-113.89)	112.92 (112.05-113.78)	0.5547
model 2	113.13 (112.10-114.15)	113.70 (112.73-114.68)	113.66 (112.73-114.59)	113.43 (112.46-114.40)	0.7360
**Diastolic blood pressure (mmHg)**					
model 1	74.80 (74.17-75.42)	75.15 (74.57-75.73)	74.94 (74.44-75.44)	75.00 (74.46-75.53)	0.8297
model 2	74.99 (74.31-75.67)	75.28 (74.61-75.96)	75.08 (74.49-75.67)	75.13 (74.53-75.73)	0.9250
**Triglyceride (mg/dL)**					
model 1	104.82 (100.69-108.95)	108.62 (104.40-112.84)	108.08 (104.01-112.15)	110.19 (105.70-114.68)	0.1335
model 2	106.86 (102.17-111.54)	109.78 (105.21-114.36)	109.60 (104.95-114.24)	111.89 (106.63-117.14)	0.1355
**Hight-density lipoprotein (mg/dL)**					
model 1	57.76 (56.91-58.61)	57.04 (56.32-57.76)	56.55 (55.86-57.23)	55.95 (55.23-56.66)	0.0006
model 2	57.47 (56.60-58.35)	56.81 (56.07-57.56)	56.37 (55.65-57.10)	55.78 (55.00-56.56)	0.0012
**Total cholesterol (mg/dL)**					
model 1	200.70 (198.49-202.90)	199.46 (197.45-201.47)	199.19 (197.43-200.94)	197.70 (195.65-199.75)	0.0480
model 2	201.35 (198.93-203.76)	200.22 (198.03-202.41)	199.77 (197.78-201.76)	198.37 (196.06-200.68)	0.0427
**TG/HDL**					
model 1	2.03 (1.93-2.14)	2.14 (2.03-2.25)	2.15 (2.04-2.26)	2.27 (2.13-2.40)	0.0130
model 2	2.08 (1.95-2.20)	2.16 (2.04-2.28)	2.18 (2.06-2.31)	2.30 (2.14-2.46)	0.0143
**Fasting blood glucose (mg/dL)**					
model 1	95.34 (94.47-96.21)	95.36 (94.64-96.08)	95.65 (94.86-96.43)	95.32 (94.51-96.13)	0.9193
model 2	95.54 (94.54-96.54)	95.45 (94.54-96.35)	95.75 (94.75-96.74)	95.46 (94.50-96.41)	0.9755
**HbA1c (%)**					
model 1	5.55 (5.52-5.58)	5.52 (5.50-5.54)	5.53 (5.50-5.55)	5.51 (5.48-5.53)	0.0752
model 2	5.56 (5.52-5.60)	5.53 (5.50-5.56)	5.53 (5.50-5.57)	5.51 (5.48-5.55)	0.0590

Abbreviations: LS-means, least-squares means; CIs, confidence intervals; Q, quartile; TG/HDL, triglyceride to HDL-cholesterol ratio: HbA1c, hemoglobin A1C.
model 1 adjusted for age (year, continuous) and energy intake (kcal, continuous)
model 2 additionally adjusted for household income (low, middle-low, middle-high, high), smoking status (never, past, ever), marriage status (married, unmarried), education level (elementary school or below, middle school, high school, college or above), prevalence of monthly alcohol use(No, Yes), physical activity (No, Yes), menopause status (No, Yes)

Table F. Least-squares means (95% confidence intervals) of health outcome according to the Quartiles of EAT-Lancet diet score in women aged 65 and above

	Quartiles of the EAT-Lancet diet score				
**LS-means (95% CIs)**	**Q1 (n=321)**	**Q2 (n=651)**	**Q3 (n=1,010)**	**Q4 (n=1,293)**	**p for trend**
**Body mass index (kg/m²)**					
model 1	25.25 (24.32-26.18)	25.06 (24.35-25.76)	25.17 (24.47-25.88)	25.31 (24.58-26.04)	0.4011
model 2	25.21 (24.28-26.13)	24.98 (24.21-25.74)	25.06 (24.30-25.83)	25.22 (24.44-26.00)	0.4795
**Waist circumferences (cm)**					
model 1	83.60 (81.17-86.04)	83.70 (81.71-85.69)	83.99 (82.01-85.97)	84.43 (82.42-86.44)	0.1059
model 2	83.98 (81.49-86.47)	84.02 (81.85-86.20)	84.25 (82.08-86.41)	84.71 (82.55-86.87)	0.1318
**Systolic blood pressure (mmHg)**					
model 1	117.13 (112.98-121.28)	114.72 (110.85-118.59)	116.05 (112.28-119.83)	114.36 (110.60-118.13)	0.0838
model 2	115.53 (111.18-119.88)	112.97 (108.81-117.12)	114.26 (110.24-118.28)	112.52 (108.50-116.54)	0.0503
**Diastolic blood pressure (mmHg)**					
model 1	82.35 (80.11-84.59)	82.06 (79.89-84.23)	81.86 (79.75-83.96)	81.05 (78.92-83.18)	0.0175
model 2	81.99 (79.54-84.45)	81.56 (79.11-84.01)	81.31 (78.93-83.68)	80.49 (78.10-82.87)	0.0076
**Triglyceride (mg/dL)**					
model 1	118.96 (103.16-134.75)	129.20 (112.03-146.36)	123.30 (109.24-137.35)	133.82 (119.33-148.31)	0.0085
model 2	124.91 (107.75-142.08)	134.52 (115.91-153.13)	129.01 (113.15-144.87)	139.27 (122.40-156.13)	0.0094
**Hight-density lipoprotein (mg/dL)**					
model 1	57.89 (55.03-60.74)	58.16 (55.59-60.73)	57.38 (54.93-59.84)	56.54 (54.21-58.87)	0.0143
model 2	57.11 (54.12-60.11)	57.52 (54.69-60.34)	56.74 (53.98-59.50)	55.96 (53.36-58.57)	0.0236
**Total cholesterol (mg/dL)**					
model 1	215.43 (206.77-224.08)	218.36 (209.99-226.73)	216.12 (208.20-224.04)	214.79 (206.98-222.60)	0.2640
model 2	213.16 (203.84-222.48)	216.47 (207.43-225.51)	214.22 (205.38-223.06)	213.04 (204.62-221.47)	0.3433
**TG/HDL**					
model 1	2.19 (1.72-2.66)	2.41 (1.91-2.92)	2.29 (1.89-2.70)	2.54 (2.12-2.96)	0.0266
model 2	2.36 (1.85-2.86)	2.56 (2.01-3.11)	2.45 (1.99-2.90)	2.68 (2.19-3.18)	0.0338
**Fasting blood glucose (mg/dL)**					
model 1	98.92 (95.25-102.58)	97.80 (94.24-101.36)	96.72 (93.46-99.98)	96.77 (93.31-100.23)	0.0387
model 2	103.22 (98.28-108.16)	102.48 (97.32-107.64)	101.41 (96.63-106.19)	101.44 (96.51-106.38)	0.0582
**HbA1c (%)**					
model 1	5.81 (5.70-5.93)	5.81 (5.70-5.92)	5.80 (5.70-5.91)	5.80 (5.70-5.91)	0.7356
model 2	5.91 (5.76-6.06)	5.92 (5.76-6.07)	5.91 (5.76-6.06)	5.91 (5.75-6.06)	0.8157

Abbreviations: LS-means, least-squares means; CIs, confidence intervals; Q, quartile; TG/HDL, triglyceride to HDL-cholesterol ratio: HbA1c, hemoglobin A1C.
model 1 adjusted for age (year, continuous) and energy intake (kcal, continuous)
model 2 additionally adjusted for household income (low, middle-low, middle-high, high), smoking status (never, past, ever), marriage status (married, unmarried), education level (elementary school or below, middle school, high school, college or above), prevalence of monthly alcohol use(No, Yes), physical activity (No, Yes), menopause status (No, Yes)
