# Supplementary material for: Association of the planetary health diet score with obesity, high blood pressure, dyslipidemia, and cardiometabolic risk markers: Using data from the 2016–2020 Korea National Health and Nutrition Examination Survey
Source: PLoS One. 2026 Jun 8;21(6):e0350821. doi: 10.1371/journal.pone.0350821 (PMC13245749; doi:10.1371/journal.pone.0350821)
Supplement: S3 Table — (PDF) [file pone.0350821.s003.pdf]

Table A. Odds ratios (95% confidence intervals) of health outcome according to the Quartiles of EAT-Lancet diet score in men age 19~39

	ORs (95% CIs) according to the quartiles EAT-Lancet diet score				
**ORs (95% CIs)**	**Q1 (n=1,435)**	**Q2 (n=983)**	**Q3 (n=674)**	**Q4 (n=314)**	***p* for trend**
**Body mass index (≥25kg/m²)**					
model 1	1	1.10 (0.92-1.32)	0.99 (0.80-1.22)	0.79 (0.60-1.04)	0.2459
model 2	1	1.10 (0.92-1.32)	0.99 (0.81-1.23)	0.79 (0.61-1.04)	0.2673
**Waist circumferences (≥90cm)**					
model 1	1	1.06 (0.88-1.28)	1.08 (0.86-1.36)	0.94 (0.71-1.26)	0.8744
model 2	1	1.07 (0.89-1.29)	1.10 (0.87-1.38)	0.95 (0.71-1.27)	0.8034
**Blood pressure (Systolic blood pressure ≥130mmHg or Diastolic blood pressure ≥85mmHg)**					
model 1	1	0.90 (0.72-1.12)	1.03 (0.82-1.30)	0.89 (0.64-1.23)	0.7195
model 2	1	0.91 (0.73-1.14)	1.02 (0.80-1.29)	0.88 (0.63-1.22)	0.6532
**Triglyceride (≥150mg/dL)**					
model 1	1	0.93 (0.76-1.15)	0.91 (0.73-1.14)	0.89 (0.66-1.21)	0.3349
model 2	1	0.95 (0.77-1.16)	0.93 (0.74-1.16)	0.92 (0.68-1.26)	0.4642
**High-density lipoprotein (<40mg/dL)**					
model 1	1	1.02 (0.80-1.30)	0.90 (0.69-1.18)	1.02 (0.72-1.45)	0.7213
model 2	1	1.03 (0.81-1.31)	0.91 (0.69-1.19)	1.01 (0.71-1.44)	0.7372
**Low-density lipoprotein (≥130mg/dL)**					
model 1	1	0.96 (0.78-1.18)	1.03 (0.82-1.29)	1.01 (0.75-1.36)	0.8488
model 2	1	0.96 (0.78-1.18)	1.03 (0.82-1.30)	1.01 (0.75-1.36)	0.8449
**Total cholesterol (≥200mg/dL)**					
model 1	1	0.95 (0.78-1.15)	1.09 (0.88-1.36)	1.01 (0.77-1.34)	0.6147
model 2	1	0.95 (0.78-1.16)	1.10 (0.88-1.38)	1.04 (0.78-1.37)	0.5233
**TG/HDL (≥3)**					
model 1	1	1.07 (0.88-1.30)	0.94 (0.75-1.18)	0.99 (0.74-1.33)	0.7388
model 2	1	1.09 (0.89-1.32)	0.96 (0.77-1.20)	1.02 (0.76-1.37)	0.9106
**Fasting blood glucose (≥100mg/dL)**					
model 1	1	1.03 (0.82-1.30)	0.85 (0.66-1.10)	1.09 (0.78-1.53)	0.7670

model 2	1	1.04 (0.82-1.31)	0.86 (0.66-1.11)	1.11 (0.79-1.56)	0.8407
**HbA1c (≥5.7%)**					
model 1	1	1.12 (0.86-1.45)	1.06 (0.81-1.40)	0.99 (0.69-1.41)	0.8308
model 2	1	1.15 (0.88-1.50)	1.08 (0.82-1.43)	0.97 (0.67-1.40)	0.8392

Abbreviations: ORs, odds ratios; CIs, confidence intervals; Q, quartile; TG/HDL, triglyceride to HDL-cholesterol ratio: HbA1c, hemoglobin A1C.
model 1 adjusted for age (year, continuous) and energy intake (kcal, continuous)
model 2 additionally adjusted for household income (low, middle-low, middle-high, high), smoking status (never, past, ever), marriage status (married, unmarried), education level (elementary school or below, middle school, high school, college or above), prevalence of monthly alcohol use(No, Yes), physical activity (No, Yes)

Table B. Odds ratios (95% confidence intervals) of health outcome according to the Quartiles of EAT-Lancet diet score in men age 40~64

ORs (95% CIs)	ORs (95% CIs) according to the quartiles EAT-Lancet diet score				
	Q1 (n=1,270)	Q2 (n=1,306)	Q3 (n=1,327)	Q4 (n=962)	*p* for trend
**Body mass index (≥25kg/m²)**					
model 1	1	0.96 (0.81-1.14)	1.04 (0.87-1.24)	0.88 (0.73-1.06)	0.4248
model 2	1	0.96 (0.80-1.14)	1.03 (0.86-1.23)	0.86 (0.71-1.04)	0.3036
**Waist circumferences (≥90cm)**					
model 1	1	0.89 (0.75-1.06)	0.91 (0.76-1.09)	0.83 (0.68-1.01)	0.0964
model 2	1	0.89 (0.75-1.06)	0.90 (0.75-1.08)	0.82 (0.68-1.00)	0.0730
**Blood pressure (Systolic blood pressure ≥130mmHg or Diastolic blood pressure ≥85mmHg)**					
model 1	1	1.26 (1.06-1.49)	1.06 (0.89-1.27)	0.98 (0.81-1.19)	0.5882
model 2	1	1.24 (1.04-1.47)	1.02 (0.85-1.23)	0.97 (0.80-1.18)	0.4594
**Triglyceride (≥150mg/dL)**					
model 1	1	1.00 (0.84-1.20)	1.06 (0.88-1.27)	1.03 (0.85-1.25)	0.6093
model 2	1	1.02 (0.85-1.22)	1.07 (0.89-1.28)	1.08 (0.88-1.31)	0.3743
**High-density lipoprotein (<40mg/dL)**					
model 1	1	0.86 (0.71-1.06)	0.88 (0.72-1.07)	0.97 (0.78-1.21)	0.7031
model 2	1	0.90 (0.73-1.10)	0.92 (0.75-1.12)	1.01 (0.81-1.25)	0.9754
**Low-density lipoprotein (≥130mg/dL)**					
model 1	1	0.97 (0.81-1.16)	0.94 (0.79-1.13)	0.95 (0.78-1.16)	0.5495
model 2	1	0.97 (0.80-1.16)	0.94 (0.78-1.13)	0.93 (0.77-1.14)	0.4358
**Total cholesterol (≥200mg/dL)**					
model 1	1	1.03 (0.87-1.22)	1.03 (0.87-1.23)	0.99 (0.81-1.20)	0.9593
model 2	1	1.02 (0.86-1.22)	1.02 (0.86-1.22)	0.98 (0.81-1.20)	0.9080
**TG/HDL (≥3)**					
model 1	1	0.96 (0.80-1.14)	1.01 (0.84-1.20)	0.98 (0.81-1.18)	0.9537
model 2	1	0.98 (0.82-1.16)	1.02 (0.86-1.22)	1.01 (0.84-1.23)	0.7629
**Fasting blood glucose (≥100mg/dL)**					
model 1	1	1.12 (0.95-1.33)	1.16 (0.97-1.39)	1.14 (0.93-1.40)	0.1409

model 2	1	1.12 (0.94-1.33)	1.14 (0.95-1.37)	1.14 (0.93-1.39)	0.1774
**HbA1c (≥5.7%)**					
model 1	1	0.89 (0.74-1.07)	0.85 (0.71-1.02)	0.89 (0.73-1.08)	0.1554
model 2	1	0.91 (0.76-1.09)	0.86 (0.72-1.04)	0.90 (0.74-1.10)	0.2016

Abbreviations: ORs, odds ratios; CIs, confidence intervals; Q, quartile; TG/HDL, triglyceride to HDL-cholesterol ratio: HbA1c, hemoglobin A1C.
model 1 adjusted for age (year, continuous) and energy intake (kcal, continuous)
model 2 additionally adjusted for household income (low, middle-low, middle-high, high), smoking status (never, past, ever), marriage status (married, unmarried), education level (elementary school or below, middle school, high school, college or above), prevalence of monthly alcohol use(No, Yes), physical activity (No, Yes)

Table C. Odds ratios (95% confidence intervals) of health outcome according to the Quartiles of EAT-Lancet diet score in men aged 65 and above

ORs (95% CIs)	ORs (95% CIs) according to the quartiles EAT-Lancet diet score				
	Q1 (n=244)	Q2 (n=534)	Q3 (n=702)	Q4 (n=879)	*p* for trend
**Body mass index (≥25kg/m²)**					
model 1	1	0.62 (0.42-0.90)	0.77 (0.54-1.11)	0.96 (0.68-1.37)	0.1441
model 2	1	0.60 (0.41-0.88)	0.75 (0.52-1.08)	0.93 (0.65-1.33)	0.1908
**Waist circumferences (≥90cm)**					
model 1	1	0.90 (0.63-1.30)	0.97 (0.69-1.36)	1.13 (0.81-1.58)	0.1512
model 2	1	0.89 (0.62-1.29)	0.96 (0.68-1.35)	1.14 (0.81-1.59)	0.1358
**Blood pressure (Systolic blood pressure ≥130mmHg or Diastolic blood pressure ≥85mmHg)**					
model 1	1	0.84 (0.60-1.19)	0.89 (0.63-1.24)	0.80 (0.57-1.11)	0.2592
model 2	1	0.84 (0.60-1.18)	0.88 (0.62-1.23)	0.80 (0.57-1.11)	0.2728
**Triglyceride (≥150mg/dL)**					
model 1	1	0.93 (0.62-1.40)	1.20 (0.83-1.73)	0.96 (0.67-1.37)	0.9874
model 2	1	0.94 (0.64-1.40)	1.23 (0.86-1.76)	1.00 (0.71-1.42)	0.7557
**High-density lipoprotein (<40mg/dL)**					
model 1	1	1.18 (0.79-1.78)	1.05 (0.71-1.55)	1.20 (0.83-1.73)	0.5206
model 2	1	1.26 (0.83-1.90)	1.11 (0.75-1.64)	1.25 (0.86-1.81)	0.4596
**Low-density lipoprotein (≥130mg/dL)**					
model 1	1	1.21 (0.85-1.73)	0.99 (0.70-1.41)	1.04 (0.73-1.47)	0.5824
model 2	1	1.23 (0.86-1.75)	0.99 (0.70-1.40)	1.02 (0.72-1.44)	0.4529
**Total cholesterol (≥200mg/dL)**					
model 1	1	1.47 (1.03-2.12)	1.02 (0.72-1.44)	1.07 (0.76-1.51)	0.2160
model 2	1	1.48 (1.03-2.11)	1.02 (0.72-1.44)	1.07 (0.76-1.51)	0.2221
**TG/HDL (≥3)**					
model 1	1	1.05 (0.73-1.51)	1.18 (0.83-1.67)	1.07 (0.77-1.50)	0.7053
model 2	1	1.08 (0.75-1.54)	1.22 (0.87-1.72)	1.12 (0.81-1.56)	0.5071
**Fasting blood glucose (≥100mg/dL)**					
model 1	1	0.72 (0.51-1.03)	0.82 (0.60-1.12)	0.82 (0.60-1.12)	0.8331

model 2	1	0.71 (0.50-1.02)	0.81 (0.59-1.11)	0.82 (0.60-1.12)	0.8767
**HbA1c (≥5.7%)**					
model 1	1	0.85 (0.60-1.20)	1.08 (0.78-1.50)	0.85 (0.63-1.14)	0.5281
model 2	1	0.87 (0.61-1.24)	1.14 (0.82-1.58)	0.89 (0.66-1.20)	0.7434

Abbreviations: ORs, odds ratios; CIs, confidence intervals; Q, quartile; TG/HDL, triglyceride to HDL-cholesterol ratio: HbA1c, hemoglobin A1C.

model 1 adjusted for age (year, continuous) and energy intake (kcal, continuous)

model 2 additionally adjusted for household income (low, middle-low, middle-high, high), smoking status (never, past, ever), marriage status (married, unmarried), education level (elementary school or below, middle school, high school, college or above), prevalence of monthly alcohol use(No, Yes), physical activity (No, Yes)

Table D. Odds ratios (95% confidence intervals) of health outcome according to the Quartiles of EAT-Lancet diet score in women age 19~39

ORs (95% CIs)	ORs (95% CLs) according to the quartiles EAT-Lancet diet score				
	Q1 (n=1,375)	Q2 (n=1,259)	Q3 (n=1,011)	Q4 (n=522)	p for trend
**Body mass index (≥25kg/m²)**					
model 1	1	0.86 (0.69-1.07)	0.88 (0.70-1.12)	0.85 (0.64-1.14)	0.2302
model 2	1	0.84 (0.67-1.04)	0.88 (0.69-1.11)	0.83 (0.62-1.12)	0.1935
**Waist circumferences (≥90cm)**					
model 1	1	0.87 (0.68-1.11)	0.77 (0.59-1.00)	0.78 (0.55-1.11)	0.0541
model 2	1	0.84 (0.65-1.09)	0.75 (0.58-0.98)	0.75 (0.53-1.07)	0.0329
**Blood pressure (Systolic blood pressure ≥130mmHg or Diastolic blood pressure ≥85mmHg)**					
model 1	1	0.72 (0.51-1.02)	0.86 (0.60-1.25)	0.43 (0.25-0.74)	0.0117
model 2	1	0.74 (0.52-1.05)	0.89 (0.61-1.29)	0.44 (0.25-0.76)	0.0181
**Triglyceride (≥150mg/dL)**					
model 1	1	0.87 (0.64-1.17)	0.93 (0.69-1.26)	0.89 (0.62-1.29)	0.5722
model 2	1	0.86 (0.64-1.16)	0.93 (0.69-1.27)	0.88 (0.60-1.28)	0.5447
**High-density lipoprotein (<50mg/dL)**					
model 1	1	0.97 (0.80-1.17)	0.85 (0.69-1.05)	1.04 (0.80-1.34)	0.5934
model 2	1	0.91 (0.75-1.11)	0.83 (0.67-1.02)	0.98 (0.76-1.27)	0.3584
**Low-density lipoprotein (≥130mg/dL)**					
model 1	1	0.98 (0.79-1.21)	0.95 (0.76-1.20)	1.04 (0.79-1.37)	0.9815
model 2	1	0.96 (0.77-1.19)	0.95 (0.75-1.20)	1.01 (0.76-1.33)	0.8759
**Total cholesterol (≥200mg/dL)**					
model 1	1	0.95 (0.79-1.15)	0.88 (0.71-1.08)	0.86 (0.66-1.12)	0.1667
model 2	1	0.96 (0.80-1.15)	0.89 (0.72-1.09)	0.86 (0.66-1.12)	0.1738
**TG/HDL (≥3)**					
model 1	1	0.78 (0.59-1.05)	0.88 (0.66-1.18)	0.90 (0.63-1.28)	0.4995
model 2	1	0.75 (0.56-1.01)	0.87 (0.65-1.17)	0.86 (0.60-1.23)	0.3854
**Fasting blood glucose (≥100mg/dL)**					
model 1	1	0.88 (0.66-1.19)	0.87 (0.65-1.17)	1.02 (0.71-1.47)	0.8070

model 2	1	0.87 (0.64-1.17)	0.86 (0.64-1.16)	1.01 (0.70-1.46)	0.7597
**HbA1c (≥5.7%)**					
model 1	1	0.84 (0.62-1.14)	0.87 (0.64-1.18)	1.08 (0.75-1.56)	0.9689
model 2	1	0.80 (0.59-1.09)	0.86 (0.64-1.17)	1.00 (0.69-1.45)	0.8236

Abbreviations: CIs, confidence intervals; HbA1c, hemoglobin A1c; ORs, odds ratios; Q, quartile; TG/HDL, triglyceride to HDL-cholesterol ratio.
model 1 adjusted for age (year, continuous) and energy intake (kcal, continuous)
model 2 additionally adjusted for household income (low, middle-low, middle-high, high), smoking status (never, past, ever), marriage status (married, unmarried), education level (elementary school or below, middle school, high school, college or above), prevalence of monthly alcohol use(No, Yes), physical activity (No, Yes), menopause status (No, Yes)

Table E. Odds ratios (95% confidence intervals) of health outcome according to the Quartiles of EAT-Lancet diet score in women age 40~64

ORs (95% CIs)	ORs (95% CLs) according to the quartiles EAT-Lancet diet score				
	Q1 (n=1,268)	Q2 (n=1,813)	Q3 (n=2,137)	Q4 (n=2,046)	p for trend
**Body mass index (≥25kg/m²)**					
model 1	1	0.97 (0.81-1.17)	1.04 (0.87-1.24)	0.98 (0.82-1.18)	0.9524
model 2	1	0.94 (0.79-1.13)	1.01 (0.84-1.21)	0.95 (0.79-1.14)	0.8014
**Waist circumferences (≥90cm)**					
model 1	1	0.99 (0.82-1.21)	1.05 (0.87-1.28)	0.97 (0.80-1.18)	0.8985
model 2	1	0.96 (0.79-1.16)	1.02 (0.84-1.24)	0.94 (0.77-1.14)	0.6889
**Blood pressure (Systolic blood pressure ≥130mmHg or Diastolic blood pressure ≥85mmHg)**					
model 1	1	1.19 (0.97-1.48)	1.11 (0.90-1.36)	1.11 (0.90-1.36)	0.6216
model 2	1	1.18 (0.96-1.47)	1.10 (0.89-1.35)	1.09 (0.89-1.34)	0.7166
**Triglyceride (≥150mg/dL)**					
model 1	1	1.07 (0.85-1.34)	1.00 (0.81-1.23)	1.07 (0.87-1.32)	0.7190
model 2	1	1.04 (0.83-1.31)	0.98 (0.79-1.21)	1.05 (0.85-1.30)	0.7918
**High-density lipoprotein (<50mg/dL)**					
model 1	1	1.07 (0.90-1.28)	1.19 (0.99-1.42)	1.18 (0.99-1.41)	0.0374
model 2	1	1.06 (0.89-1.28)	1.17 (0.98-1.40)	1.17 (0.98-1.40)	0.0549
**Low-density lipoprotein (≥130mg/dL)**					
model 1	1	0.95 (0.80-1.12)	0.99 (0.85-1.16)	0.86 (0.73-1.01)	0.1049
model 2	1	0.96 (0.81-1.13)	0.99 (0.85-1.15)	0.86 (0.73-1.01)	0.0932
**Total cholesterol (≥200mg/dL)**					
model 1	1	0.90 (0.77-1.06)	0.92 (0.79-1.07)	0.79 (0.67-0.93)	0.0115
model 2	1	0.90 (0.77-1.06)	0.92 (0.79-1.07)	0.79 (0.67-0.94)	0.0111
**TG/HDL (≥3)**					
model 1	1	1.08 (0.87-1.34)	1.09 (0.89-1.33)	1.16 (0.95-1.42)	0.1539
model 2	1	1.06 (0.85-1.32)	1.07 (0.87-1.31)	1.14 (0.94-1.40)	0.1946
**Fasting blood glucose (≥100mg/dL)**					
model 1	1	1.15 (0.94-1.40)	1.14 (0.95-1.37)	1.11 (0.92-1.34)	0.4137
model 2	1	1.13 (0.93-1.38)	1.13 (0.94-1.36)	1.11 (0.92-1.34)	0.3914
**HbA1c (≥5.7%)**					
model 1	1	0.94 (0.78-1.12)	0.94 (0.79-1.11)	0.83 (0.70-1.00)	0.0494
model 2	1	0.94 (0.79-1.13)	0.93 (0.79-1.11)	0.83 (0.69-0.99)	0.0433

Abbreviations: CIs, confidence intervals; HbA1c, hemoglobin A1c; ORs, odds ratios; Q, quartile; TG/HDL, triglyceride to HDL-cholesterol ratio.
model 1 adjusted for age (year, continuous) and energy intake (kcal, continuous)
model 2 additionally adjusted for household income (low, middle-low, middle-high, high), smoking status (never, past, ever), marriage status (married, unmarried), education level (elementary school or below, middle school, high school, college or above), prevalence of monthly alcohol use(No, Yes), physical activity (No, Yes), menopause status (No, Yes)

Table F. Odds ratios (95% confidence intervals) of health outcome according to the Quartiles of EAT-Lancet diet score in women aged 65 and above

ORs (95% CIs)	ORs (95% CLs) according to the quartiles EAT-Lancet diet score				
	Q1 (n=321)	Q2 (n=651)	Q3 (n=1,010)	Q4 (n=1,293)	p for trend
**Body mass index (≥25kg/m²)**					
model 1	1	0.91 (0.66-1.28)	0.91 (0.66-1.24)	0.99 (0.73-1.35)	0.7401
model 2	1	0.90 (0.65-1.26)	0.88 (0.64-1.20)	0.96 (0.71-1.30)	0.9357
**Waist circumferences (≥90cm)**					
model 1	1	0.97 (0.70-1.35)	0.96 (0.70-1.30)	1.05 (0.78-1.42)	0.5328
model 2	1	0.96 (0.69-1.33)	0.94 (0.69-1.28)	1.03 (0.76-1.38)	0.6404
**Blood pressure (Systolic blood pressure ≥130mmHg or Diastolic blood pressure ≥85mmHg)**					
model 1	1	0.61 (0.45-0.84)	0.76 (0.57-1.00)	0.61 (0.46-0.80)	0.0119
model 2	1	0.60 (0.44-0.83)	0.74 (0.56-0.99)	0.60 (0.45-0.79)	0.0105
**Triglyceride (≥150mg/dL)**					
model 1	1	1.29 (0.90-1.84)	0.99 (0.69-1.43)	1.38 (0.98-1.94)	0.1268
model 2	1	1.26 (0.87-1.81)	0.98 (0.68-1.41)	1.34 (0.95-1.90)	0.1614
**High-density lipoprotein (<50mg/dL)**					
model 1	1	0.91 (0.67-1.24)	0.95 (0.70-1.28)	1.17 (0.88-1.55)	0.0549
model 2	1	0.91 (0.67-1.25)	0.95 (0.70-1.29)	1.16 (0.86-1.55)	0.0709
**Low-density lipoprotein (≥130mg/dL)**					
model 1	1	1.07 (0.78-1.46)	1.02 (0.76-1.37)	0.94 (0.71-1.25)	0.3521
model 2	1	1.09 (0.80-1.49)	1.04 (0.77-1.39)	0.95 (0.71-1.27)	0.3615
**Total cholesterol (≥200mg/dL)**					
model 1	1	1.10 (0.81-1.49)	0.93 (0.69-1.25)	0.91 (0.69-1.21)	0.1514
model 2	1	1.13 (0.84-1.53)	0.96 (0.71-1.28)	0.93 (0.70-1.24)	0.1910
**TG/HDL (≥3)**					
model 1	1	1.00 (0.71-1.42)	1.00 (0.70-1.42)	1.29 (0.93-1.78)	0.0305
model 2	1	0.97 (0.68-1.39)	0.98 (0.68-1.40)	1.24 (0.89-1.73)	0.0480
**Fasting blood glucose (≥100mg/dL)**					
model 1	1	0.85 (0.62-1.16)	0.78 (0.58-1.05)	0.73 (0.56-0.96)	0.0227
model 2	1	0.86 (0.63-1.17)	0.79 (0.59-1.06)	0.74 (0.56-0.97)	0.0269
**HbA1c (≥5.7%)**					
model 1	1	0.81 (0.58-1.13)	0.82 (0.60-1.13)	0.74 (0.55-1.00)	0.0680
model 2	1	0.84 (0.60-1.16)	0.85 (0.62-1.17)	0.76 (0.56-1.03)	0.0989

Abbreviations: CIs, confidence intervals; HbA1c, hemoglobin A1c; ORs, odds ratios; Q, quartile; TG/HDL, triglyceride to HDL-cholesterol ratio.
model 1 adjusted for age (year, continuous) and energy intake (kcal, continuous)
model 2 additionally adjusted for household income (low, middle-low, middle-high, high), smoking status (never, past, ever), marriage status (married, unmarried), education level (elementary school or below, middle school, high school, college or above), prevalence of monthly alcohol use(No, Yes), physical activity (No, Yes), menopause status (No, Yes)
